# An engineered antibody binds a distinct epitope and is a potent inhibitor of murine and human VISTA

**DOI:** 10.1038/s41598-020-71519-4

**Published:** 2020-09-16

**Authors:** Nishant Mehta, Sainiteesh Maddineni, Ryan L. Kelly, Robert B. Lee, Sean A. Hunter, John L. Silberstein, R. Andres Parra Sperberg, Caitlyn L. Miller, Amanda Rabe, Louai Labanieh, Jennifer R. Cochran

**Affiliations:** 1 Department of Bioengineering, Stanford University, Stanford, CA 94305 USA; 2 xCella Biosciences, Menlo Park, CA 94025 USA; 3 Department of Chemical Engineering, Stanford University, Stanford, CA 94305 USA; 4 Cancer Biology Program, Stanford University School of Medicine, Stanford, CA 94305 USA; 5 Immunology Program, Stanford University School of Medicine, Stanford, CA 94305 USA

**Keywords:** Drug development, Immunotherapy, Protein engineering, Screening, Tumour immunology, Cancer immunotherapy

## Abstract

V-domain immunoglobulin (Ig) suppressor of T cell activation (VISTA) is an immune checkpoint that maintains peripheral T cell quiescence and inhibits anti-tumor immune responses. VISTA functions by dampening the interaction between myeloid cells and T cells, orthogonal to PD-1 and other checkpoints of the tumor-T cell signaling axis. Here, we report the use of yeast surface display to engineer an anti-VISTA antibody that binds with high affinity to mouse, human, and cynomolgus monkey VISTA. Our anti-VISTA antibody (SG7) inhibits VISTA function and blocks purported interactions with both PSGL-1 and VSIG3 proteins. SG7 binds a unique epitope on the surface of VISTA, which partially overlaps with other clinically relevant antibodies. As a monotherapy, and to a greater extent as a combination with anti-PD1, SG7 slows tumor growth in multiple syngeneic mouse models. SG7 is a promising clinical candidate that can be tested in fully immunocompetent mouse models and its binding epitope can be used for future campaigns to develop species cross-reactive inhibitors of VISTA.

## Introduction

In many cancers, immune cells capable of tumor clearance infiltrate the tissue but are suppressed or directed towards inactivity. Antibodies known as checkpoint inhibitors can bolster the anti-tumor immune response by blocking immune regulation between T cells, antigen-presenting cells (APCs), and tumors, slowing down progression or even clearing the tumor. These antibody therapeutics have emerged as effective treatments for patients who are refractory to chemotherapy, and as a first-in-line therapy for multiple cancer types.

VISTA, or V-domain Ig Suppressor of T Cell Activation, is an immunoregulatory protein expressed at high levels on myeloid-derived cells such as CD11b ^+^ monocytes and CD11c^ +^ dendritic cells, to a lesser extent on CD4 ^+^ and CD8^ +^ lymphocytes, and in some cases on non-hematopoietic tumor cells^1^. As a natural homeostatic checkpoint that prevents excessive immune function, VISTA is thought to play a role in maintaining the quiescent state of CD4 ^+^ T cells; agonism of this pathway is thought to lead to increased T cell tolerance^2^. In the context of cancer, VISTA is upregulated on immunosuppressive tumor infiltrating leukocytes such as inhibitory regulatory T cells (Tregs) and myeloid-derived suppressor cells (MDSCs)^3^. The presence of VISTA in the tumor microenvironment hinders effective T cell responses and has been implicated in a number of human cancers including prostate^4^, colon^5^, skin^6^, pancreatic^7^, and lung^8^. While its role in dampening immune responses is evident, the mechanism by which VISTA functions is still under investigation.

Adding to the lack of mechanistic clarity is evidence that VISTA functions as both a ligand and a receptor. As a ligand, VISTA is expressed on APCs and binds an unknown receptor on T cells to inhibit downstream T cell activation^1,9^. As a receptor, VISTA is expressed on T cells and transduces intracellular inhibitory signals after ligand binding to curtail T cell activity^10,11^. VISTA is considered a member of the B7 protein family due to its proposed function and its immunoglobulin type fold. Out of all B7 family members, VISTA is most similar to PD-L1 by sequence alignment; however, VISTA contains only a single Ig-like V domain, similar to B7 checkpoint receptors CTLA-4 and PD-1. The structure of the extracellular domain of human VISTA was recently elucidated^12^, highlighting its two additional disulfide bonds and protruding C–C’ loop compared to other B7 family members. Ambiguity regarding the native binding partner of VISTA has also been an obstacle in understanding VISTA function. Two independent protein interaction screens identified VSIG3 (also called IGSF11), a ligand involved in cell adhesion, as a VISTA binding partner^13,14^. More recently, a pH-dependent binding interaction was identified between VISTA and PSGL-1^15^, a receptor expressed on leukocytes that plays a role in immune cell trafficking.

Multiple approaches are being taken to develop VISTA inhibitors. A small molecule purported to inhibit PD-L1, PD-L2, and VISTA is under evaluation by Curis in phase 1 trials (CA-170; NCT02812875). A clinical trial involving an anti-human VISTA antibody of the human IgG1 isotype developed by Janssen/ImmuNext (VSTB112; NCT02671955) was terminated, however, the molecule is now being pursued by Curis (CI-8993). Additionally, anti-VISTA antibodies from Bristol-Myers Squibb (BMS767) and Hummingbird Biosciences (HMBD-002) are in preclinical development. All three of these antibodies have been generated from animal immunization efforts. In this study, we used in vitro yeast screening methods to identify and engineer a cross-reactive antibody that binds with high affinity to human, murine, and cyno VISTA and is a potent inhibitor of its function. We used yeast surface display to map the VISTA binding epitope of this antibody and show that it is overlapping, but distinct from antibodies VSTB112 and BMS767, which bind only to human VISTA. We further demonstrate the ability of our antibody to block both VSIG3 and PSGL-1 binding interactions to VISTA and to delay tumor growth in several syngeneic tumor models. The strategy outlined here for cross-reactive antibody engineering, along with the elucidation of potential binding epitopes for VISTA antibodies, as well as PSGL-1 and VSIG3, will bolster efforts for continued therapeutic development.

## Results

### Engineering a cross-reactive anti-VISTA antibody using yeast surface display

**Figure 1:**
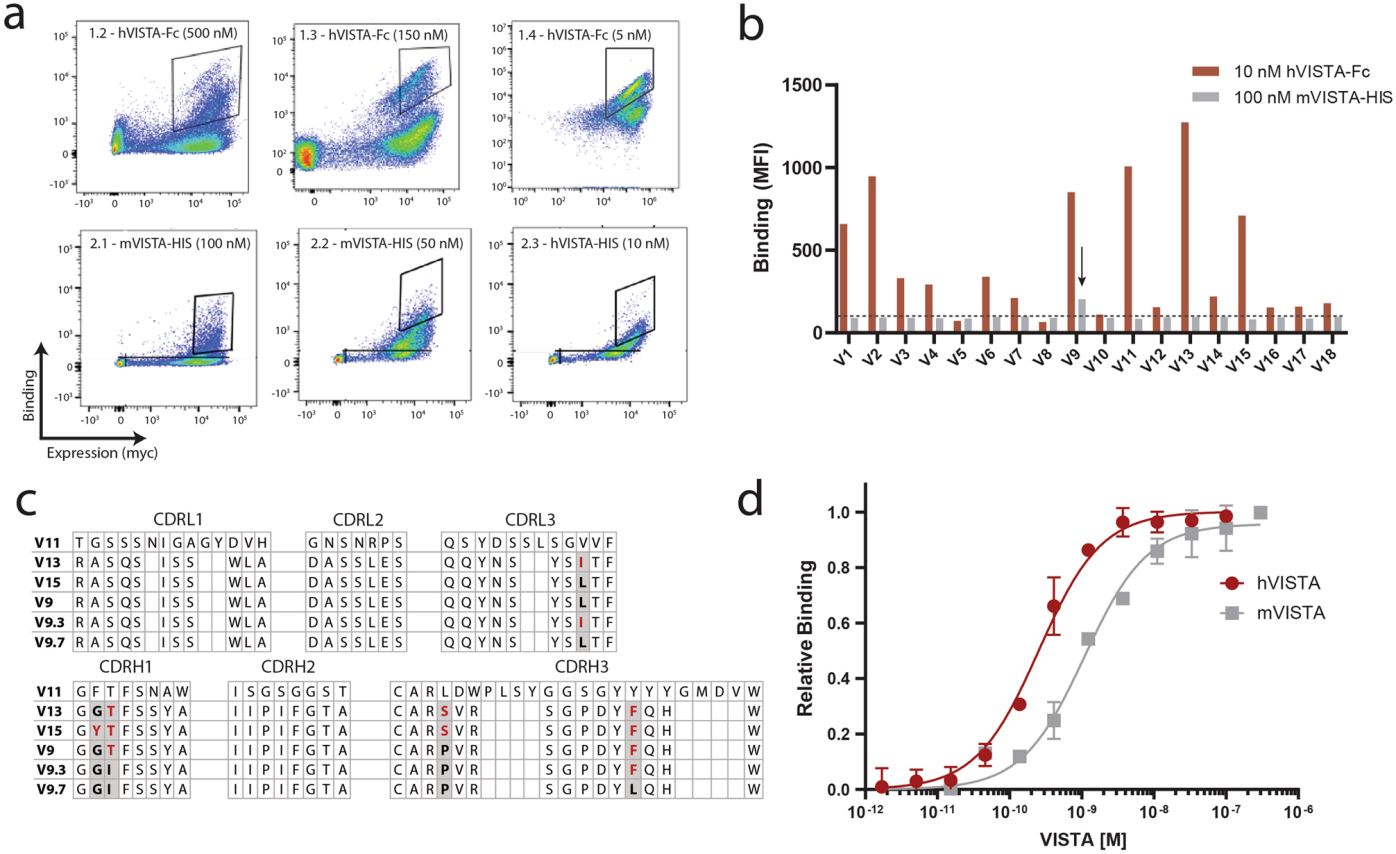
Engineering a cross-reactive anti-VISTA antibody. (**a**) Library screening progression used to isolate scFv variants that bound human VISTA (Round 1) and mouse VISTA (Round 2). Flow cytometry gates used for screening are shown on dot plots of individual sorts. X-axis depicts expression of scFv on the yeast cell surface, y-axis depicts VISTA binding, measured by antibodies against c-myc and VISTA, respectively. (**b**) Binding intensity to human VISTA-Fc (red) and mouse VISTA-His (gray) of individual scFv clones isolated after Round 1 of screening. The V9 clone displayed above background binding signal to mouse VISTA-His. (**c**) Sequence comparison of scFv clones after the first round (V9, V11, V13, V15) and after the second round (V9.3 and V9.7) of sorting. Amino acid differences from final V9.7 clone are highlighted in red. (**d**) Full kinetic binding curves of SG7 (antibody form of top clone V9.7) against human VISTA-His and mouse VISTA-His monomers, as measured by KinExA. Mean ± standard deviation of duplicate measurements are shown for D.

Unlike immunization-based approaches for antibody discovery, in vitro library strategies allow for precise and facile screening against antigens derived from murine, human, or cyno sources to enable development of a species cross-reactive VISTA binder^16^. The xEmplar library, developed by xCella Biosciences, consists of ~ 10^9^ scFv mutants that are individually fused to cell wall-anchored proteins for yeast surface display. Expression levels of scFv clones and their corresponding binding to soluble mouse or human VISTA (mVISTA or hVISTA) can be detected simultaneously using fluorescent antibodies (Supplementary Fig. S1A). For an initial round of screening, the scFv library was incubated with human VISTA coupled to magnetic beads, and the pool of bead-bound yeast cells was collected. This process of magnetic-activated cell sorting (MACS) was used to decrease library size to allow for full diversity coverage in subsequent screening steps. The partially enriched library was then screened using iterative rounds of fluorescence-activated cell sorting (FACS) against decreasing concentrations of recombinant human VISTA-Fc fusion (hVISTA-Fc) (Fig. 1a, top). The yeast pool from the fourth round of sorting was sequenced and 18 distinct clones were identified and displayed individually on yeast. To assess species cross-reactivity, clones were tested for binding to 10 nM hVISTA-Fc or 100 nM hexahistidine-tagged mouse VISTA (mVISTA-His) under equilibrium conditions (Fig. 1b). While all clones showed detectable binding to hVISTA, only one clone (V9) demonstrated binding signal above background, albeit weak, to mVISTA. Clone V9 was also one of the top binders to hVISTA, making it an ideal choice for further affinity maturation against mVISTA.

A second scFv library was designed, heavily weighted towards clone V9 while including other enriched clones from sort 4 for an expanded search space. Pooled DNA was subjected to error-prone PCR across the entire scFv, targeting 2–3 amino acid mutations per gene. The resulting library was transformed into yeast and screened by FACS for binding to mVISTA-His (Fig. 1a, bottom). Significant enrichment against 50 nM mVISTA-His was observed in the second round of sorting and the top 10% of the population was collected. A final screen against hVISTA-His, using kinetic off-rate conditions, was performed to isolate only clones that retained high affinity binding to human VISTA. The yeast population was sequenced and two predominant clones, V9.3 and V9.7, were identified. Binding of yeast-displayed clones V9, V9.3, and V9.7 was measured against hVISTA-Fc and mVISTA-His (Supplementary Fig. S1B, S1C). Clone V9.7 differed from parental clone V9 by just two amino acids (Fig. 1c) and was chosen for further studies as V9.3 contained framework mutations. Yeast-displayed V9.7 bound cyno VISTA with high affinity (apparent K_d_ < 0.5 nM), similar to human VISTA (Supplementary Fig. S2), which is expected given the > 95% sequence identity between the extracellular domains.

The VH and VL genes from scFv clone V9.7 were converted to full length mIgG2a heavy and light chains and expressed as soluble antibody in HEK cells (called SG7 in antibody form). Titration binding curves between SG7 and monovalent antigen, hVISTA-His and mVISTA-His, were measured using the Kinetic Exclusion Assay (KinExA). SG7 bound to hVISTA and mVISTA with dissociation constants (K_d_) of 140 ± 130 pM and 1.0 ± 0.3 nM, respectively (Fig. 1d). This binding data demonstrates that the emergence of mutations conferring low nanomolar binding affinity to mouse VISTA does not interfere with the tight binding affinity to human VISTA observed from the parent clone. The workflow described here of screening against an antigen from one species, diversifying a weighted library, and screening against a species homolog is broadly applicable to future cross-reactive antibody engineering campaigns.

### SG7 is an inhibitor of mouse and human VISTA

**Figure 2:**
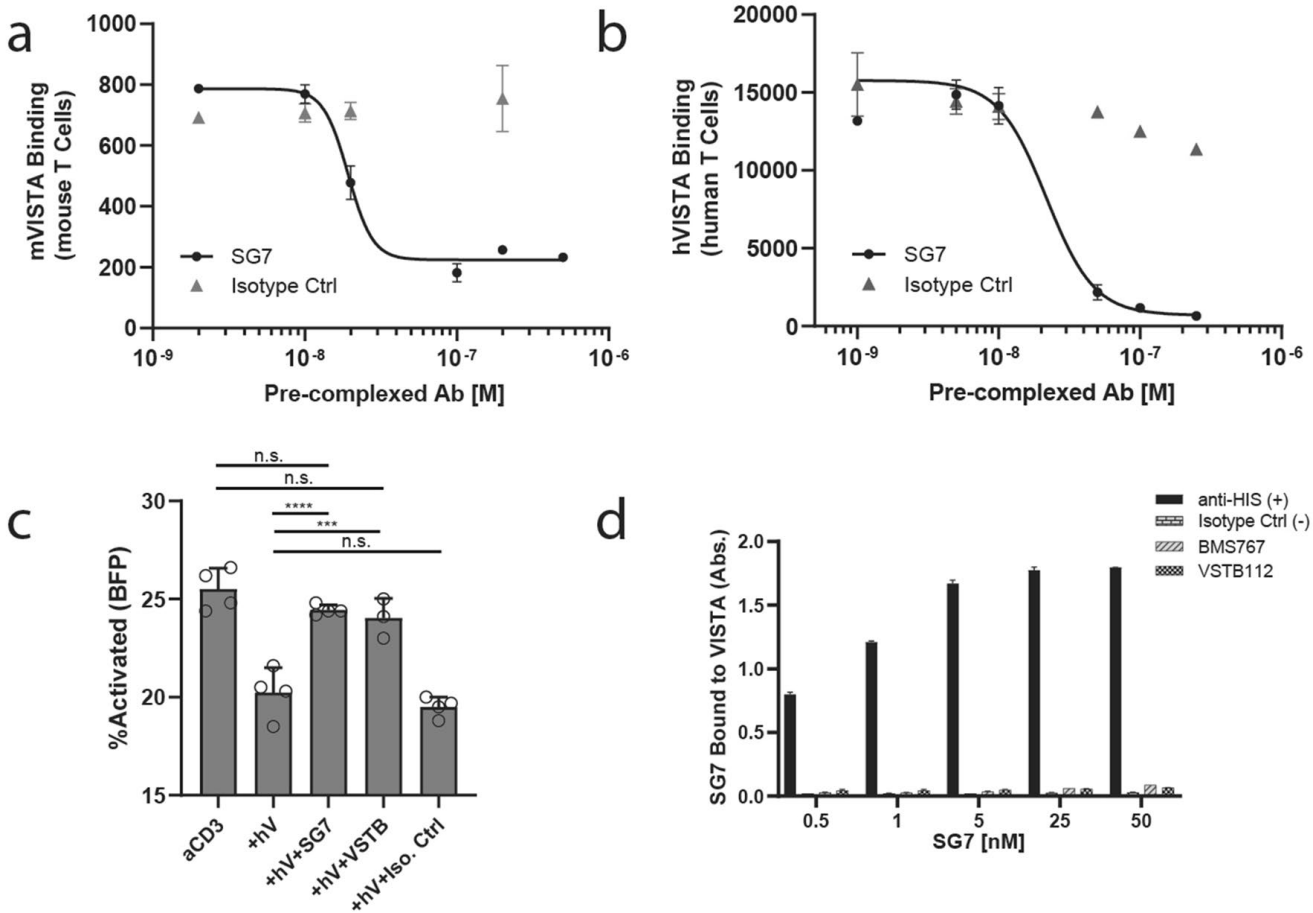
SG7 is an inhibitor of mouse and human VISTA. (**a**) Mouse VISTA-Fc binding to activated mouse T cells at pH 6.0 with increasing concentrations of pre-complexed SG7 or isotype control. (**b**) Human VISTA-Fc binding to activated human T cells at pH 6.0 with increasing concentrations of pre-complexed SG7 or isotype control. (**c**) Rescuing effect of SG7 and VSTB112 on the activation of Jurkat NFAT (BFP) T cells in the presence of human VISTA-Fc. P-values obtained by one-way ANOVA (Tukey’s multiple comparison test), ***p < .005, ****p < .0005. (**d**) Competition ELISA to test simultaneous binding of SG7 and microtiter well-coated BMS767 or VSTB112 to soluble human VISTA. Binding signal of SG7 bound to the complex of VISTA and coated antibody is shown with increasing concentrations of SG7. In this assay, only the anti-His positive control can bind simultaneously to SG7 and VISTA. Mean ± SD for triplicate measurements are shown for all panels.

We next determined the ability of SG7 to block VISTA binding to primary T cells as a direct measure of inhibition. VISTA has been shown to bind activated T cells at low pH through a histidine-dependent binding interface with PSGL-1^15^. Splenocytes from C57BL/6 mice were activated with anti-CD3/anti-CD28 beads and then incubated with mVISTA-Fc in complex with different concentrations of SG7 at pH 6.0. We found that SG7 blocks mVISTA-Fc from binding to mouse T cells in a dose-dependent manner, where > 10 nM SG7 completely blocked the interaction (Fig. 2a). Interestingly, mVISTA-Fc did not bind activated mouse T cells at physiological pH (Supplementary Fig. S3A). Human T cells (CD4^+^ and CD8^+^ cells) were activated with anti-CD3/anti-CD28 beads and then incubated with hVISTA-Fc in complex with SG7 at pH 6.0. A dose-dependent response was observed, where > 50 nM SG7 blocked the hVISTA/T cell interaction (Fig. 2b). These results demonstrate that the SG7 antibody can inhibit interactions between VISTA and T cells of both murine and human origin.

Next, a T cell activation assay with a human Jurkat T cell-NFAT reporter line was used to assess the ability of SG7 to functionally inhibit VISTA signaling. Jurkat T cells were activated with anti-CD3 in the presence of hVISTA-Fc. Consideration was taken to confirm that the addition of hVISTA-Fc did not interfere with activation through sterics or non-specific binding (Supplementary Fig. S3B). SG7 or VSTB112 (Janssen Therapeutics), used here as a positive control for VISTA inhibition, was added as a third component to measure its respective ability to block VISTA function and rescue levels of T cell activation. Both antibodies were able to restore activation to anti-CD3 only levels (Fig. 2c). An isotype control antibody, which does not bind VISTA, did not show effective inhibition.

Finally, we assessed if SG7 could bind to VISTA when co-incubated with one of two clinically relevant VISTA inhibitor antibodies, VSTB112 or BMS767 (Bristol Myers Squibb). Competitor antibodies were coated on a microtiter plate and incubated with human VISTA. SG7 was then briefly added at a range of concentrations, but was not able to bind to VISTA complexed with the BMS767 or VSTB112 plate-coated antibodies (Fig. 2d). Lack of SG7 binding even at high concentrations suggests that the binding epitope of SG7 overlaps with these antagonist antibodies. Epitope binning using a ForteBio Octet confirmed that all three antibodies cross-block each other (Supplementary Fig. S4). These data demonstrate that SG7 not only competes with other clinically relevant antibodies for VISTA binding and blocks binding to primary T cells, but also functionally inhibits VISTA for the restoration of normal T cell activation.

### SG7 binds to a distinct epitope on VISTA

**Figure 3:**
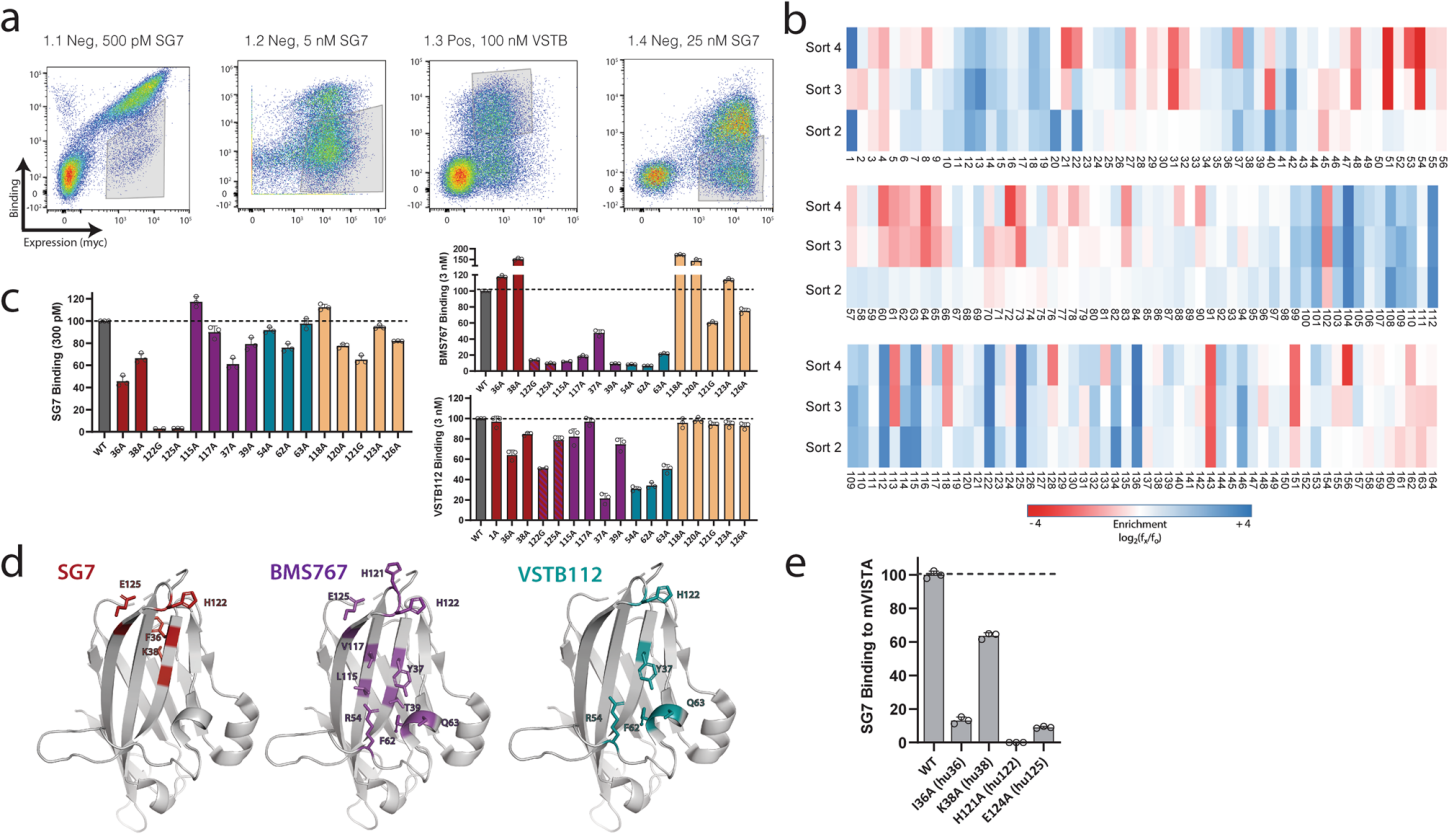
SG7 binds to a distinct epitope on VISTA. (**a**) Library screening progression used to isolate human VISTA mutants that lost binding to SG7 but retained binding to VSTB112. Flow cytometry gates used in screening are shown on dot plots of individual sorts. (**b**) Enrichment of mutations at each residue location in human VISTA obtained from NGS analysis of gated populations after each epitope mapping sort (blue—more enriched, red—less enriched). (**c**) Analysis of individual human VISTA mutations expressed on yeast. Binding intensity of each mutant to SG7, BMS767, or VSTB112 at the respective approximate dissociation constant (K_d_) of each antibody. Bars are colored based on predicted epitope (SG7-red, BMS767-purple, VSTB112-cyan, neighboring residues-beige). Binding signal of each mutant was normalized to wild-type hVISTA. (**d**) Epitopes of SG7 (red), BMS767 (purple), and VSTB112 (cyan) antibodies based on single clone binding analysis. (**e**) Analysis of individual mouse VISTA mutations displayed on yeast. Selected mutants correspond with aligned human VISTA residues (in parentheses) that make up or are near the predicted SG7 epitope. Binding intensity of each mutant to 4 nM SG7 is shown. Binding signal of each mutant was normalized to wild-type mVISTA. Mean ± SD for triplicate measurements are shown for (**c**,**e**).

We next investigated the binding epitope of SG7 to elucidate how it engages both mouse and human VISTA in contrast to the BMS767 and VSTB112 antibodies, which only bind to human VISTA. Although our data suggests that SG7 cross-blocks these antibodies and vice-versa, the residues that make up their individual epitopes must contain differences to explain the species cross-reactivity of SG7. A previously described method of fine-epitope mapping with yeast display^12,17^ was used to identify potential antibody binding residues on VISTA. A library of human VISTA mutants was generated using error-prone PCR to create an average of one amino acid mutation per gene. The library was transformed into yeast and screened using several iterative rounds of FACS (Fig. 3a). First, a ‘negative’ sort was carried out to isolate VISTA variants that were expressed on the yeast cell surface but that did not bind to 0.5 nM SG7. In sort 2, remaining mutants that had lost binding to SG7 were screened against 5 nM SG7 for increased stringency. VSTB112 binds a conformational epitope^12^ and could therefore be used as a positive control for structural integrity. Thus, to select against mutations that caused protein misfolding, the library was screened with VSTB112 in sort 3 and only clones that retained binding to 100 nM VSTB112 were collected. In sort 4, the screening stringency was again increased to enrich for clones that almost completely lost binding to 25 nM SG7. Yeast pools collected after each sort round were deep sequenced to comprehensively identify enriched clones. Enrichment of mutations at each residue after sort 2–4 was calculated based on frequency of mutation compared to the base library (Fig. 3b). Upon preliminary examination, it appeared that many distinct regions of the protein were enriched. In particular, strong enrichment was observed in residues 10–19, 100–115, 134, and 136. However, structural analysis indicates that many of these residues are heavily buried or contribute to formation of the beta barrel of the VISTA protein. Although these mutations passed through the positive sort against VSTB112, they likely modify the local structure, causing partial misfolding and preventing SG7 binding. To test this hypothesis, we analyzed four mutations that appeared buried or integral to the beta barrel (T100A, M101A, L104A, L134A) as individual VISTA point mutants displayed on yeast. All four mutations decreased binding significantly to all three antibodies, strongly suggesting that they affect the tertiary structure and are not specific to a particular epitope (Supplementary Fig. S5). We therefore proceeded to discard residues that were buried or made up the bottom beta barrel of VISTA and ranked the remaining residues by enrichment, leaving a set of four proposed residues that comprise the SG7 binding epitope: F36, K38, H122, and E125.

To compare the SG7 epitope with BMS767 and VSTB112, we tested a panel of individual alanine mutations in VISTA that encompass the predicted regions of these three antibodies (Fig. 3c). Previous epitope mapping work with BMS767 showed the importance of VISTA residues in the C and F beta strands that point towards the C–C’ loop: Y37, T39, L115, and V117 (Fig. 3c, purple bars)^15^. Earlier studies by our group demonstrated the importance of the R54, F62, and Q63 residues for binding to VSTB112 (Fig. 3c, cyan bars)^12^. We also expanded the panel to include five residues adjacent in 3D space to the mapped SG7 region to probe for locations potentially missed by the yeast screen (Fig. 3c, beige bars). Yeast-displayed clones containing single alanine mutations or wild-type (WT) VISTA were tested with concentrations of SG7, BMS767, or VSTB112 at or near the approximate dissociation constant (K_d_) of each antibody. Mutations that caused a drop below 50% of WT VISTA binding were considered critical residues and guided the predicted epitopes for each antibody (Fig. 3d). This single clone analysis suggests that: (1) SG7 and BMS767 binding epitopes share at least two residues (H122, E125) within the histidine-rich tip of the protein, (2) SG7 is the only antibody of the three tested to be significantly affected by mutations at F36 and K38, (3) BMS767 appears to bind to the histidine-rich tip as well as residues in the C–C’ loop and adjacent helix (C–C’ helix), and (4) VSTB112 and SG7 bind mostly distinct regions with potential overlap at H122. We also observed that the F36 and K38 mutations, which uniquely affect SG7, point towards the opposite face of the protein compared to the proposed binding regions of BMS767 and VSTB112, further highlighting a potentially distinct epitope.

To additionally explore residues underlying cross-reactivity, we investigated whether the four SG7 epitope residues proposed on human VISTA (F36, K38, H122, and E125) also drive the antibody interaction with mouse VISTA. Protein sequences of both species of VISTA were aligned and the four analogous mouse VISTA residues (I36, K38, H121, E124) were mutated to alanine, individually displayed on yeast, and measured for binding to SG7 by flow cytometry (Fig. 3e). The two epitope residues in the histidine rich tip (human: H122, E125; mouse: H121, E124) as well as the back-facing residues of F36 (I36 in mouse) and, to a lesser extent, K38, evidently mediate SG7 binding to mVISTA. These data suggest that SG7 has a unique antibody binding interface; it shares the histidine rich tip with BMS767 but binding is also dependent on an alternate patch in a nearby region which includes the F36 and K38 residues. The strong antibody binding dependence on these four residues across species of VISTA suggests that other VISTA antibodies targeted to this area would also be cross-reactive with mouse and human proteins.

### SG7 inhibits VISTA binding to PSGL-1 or VSIG3

**Figure 4:**
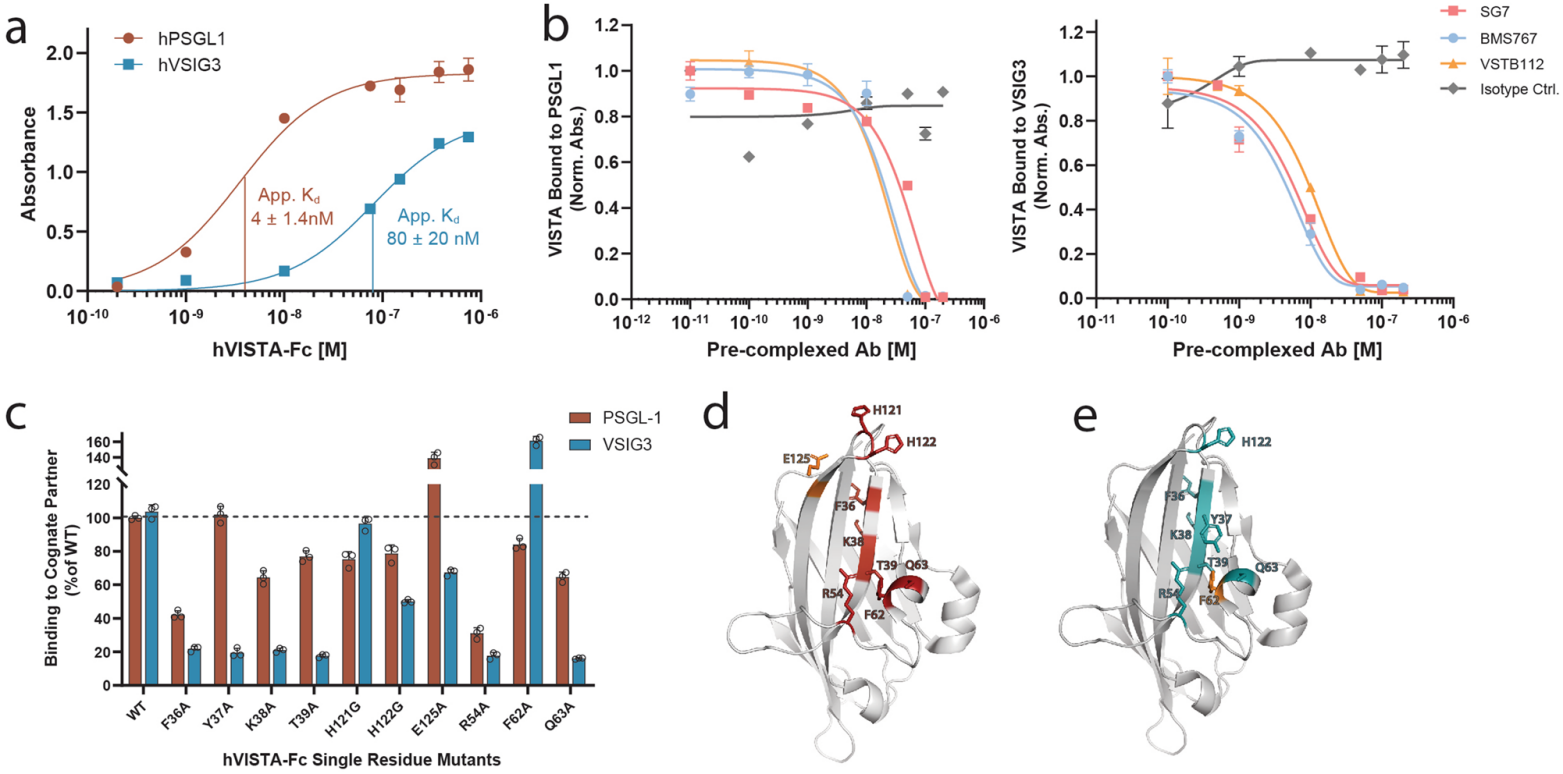
SG7 blocks VISTA binding interactions with PSGL-1 and VSIG3. (**a**) ELISA binding assay with microtiter well-coated hPSGL-1-Fc or hVSIG3-Fc incubated with human VISTA-Fc. (**b**) Competition binding ELISA with microtiter well-coated PSGL-1-Fc (left) or VSIG3-Fc (right) and increasing concentrations of SG7, BMS767, or VSTB112 in complex with 250 nM human VISTA-Fc. The binding signal of VISTA that was able to bind coated PSGL-1 or VSIG3 was detected. Competition binding ELISAs were performed at pH 6.0. (**c**) Single clone analysis of soluble VISTA-Fc mutants binding to well-coated PSGL-1-Fc or VSIG3-Fc in ELISA format. The binding signal of each VISTA-Fc mutant was normalized to wild-type VISTA binding to PSGL-1 or VSIG3, respectively. (**d,e**) Predicted epitope of VISTA binding to both cognate binding partners, PSGL-1 (red) and VSIG3 (cyan), based on single clone analysis using a panel of soluble mutants. Residue locations at which alanine mutations increased binding to PSGL-1 (**d**) or VSIG3 (**e**) are shown in orange. Mean ± SD for triplicate measurements are shown for **a**–**c**.

In addition to mapping the binding epitope of VISTA with SG7 and two clinically-relevant antibodies, we investigated the regions of interaction with two purported VISTA binding partners, PSGL-1 and VSIG3. We first characterized these ligand-receptor interactions in their soluble form via ELISA. VSIG3-Fc or PSGL-1-Fc coated on microtiter wells was incubated with different concentrations of VISTA-Fc at pH 6.0, resulting in apparent binding affinity measurements of 4 ± 1 nM for VISTA/PSGL-1 and 80 ± 20 nM for VISTA/VSIG3 (Fig. 4a). The binding interaction for PSGL-1 was heavily dependent on pH, with no measurable binding at physiological pH (Supplementary Fig. S6A). In contrast, VSIG3 had a four-fold improvement in binding affinity at physiological pH compared to pH 6.0 (Supplementary Fig. S6B). We next tested whether SG7 could affect PSGL-1 and VSIG3 binding interactions with VISTA. VISTA-Fc was pre-complexed with varying concentrations of SG7 or isotype control antibody and added to microtiter wells coated with PSGL-1-Fc or VSIG3-Fc (Fig. 4b). BMS767 and VSTB112 were used as positive controls because they have been shown to block PSGL-1 or VSIG3, respectively^12,15^. All three antibodies were able to compete with native PSGL-1/VISTA or VSIG3/VISTA binding in a similar dose-dependent manner.

A panel of solubly-expressed VISTA-Fc mutants was designed to further explore native VISTA binding interactions. Epitope residues for all three antibodies identified from yeast-displayed studies were mutated to glycine or alanine. Similar structural elements were observed for all purified VISTA mutants as confirmed by circular dichroism (Supplementary Fig. S6C). Each VISTA-Fc mutant was added to PSGL-1-Fc or VSIG3-Fc coated wells at a concentration roughly equivalent to the apparent K_d_ of the WT VISTA interaction. Binding interactions were measured at pH 6.0 for both proteins. Mutation of VISTA residues R54 and F36 significantly perturbed the interaction with PSGL-1, as defined by a > 50% drop in binding (Fig. 4c). Mutation of a number of VISTA residues partially affected PSGL-1 binding (> 20% drop): K38, T39, H121, H122, and Q63 (Fig. 4c,d). While these residues do not form an obvious contiguous surface, the histidine rich tip and the back-facing residues overlap significantly with the SG7 and BMS767 epitopes. For VSIG3, mutation of many residues significantly decreased binding: F36, Y37, K38, T39, H122, R54, and Q63 (Fig. 4c,e). The sensitivity of residues 36–39 to mutation suggest VSIG3 interacts heavily with this beta strand and is perturbed by local aberrations. Residues R54 and Q63, which both affected PSGL-1 and VSIG3 binding, likely play a role in stabilizing a helix and adjacent C–C’ loop in VISTA. Interestingly, mutations E125A and F62A increased binding to PSGL-1 and VSIG3, respectively. Binding curves of these two mutants measured via ELISA showed affinities at least two-fold greater than the WT VISTA interaction (Supplementary Fig. S6D, S6E). The increased affinity suggests that these mutations may improve local electrostatic interactions with PSGL-1 and VSIG3, and represent a potential avenue for engineering a higher affinity VISTA molecule.

### VISTA blockade reduces tumor growth in multiple syngeneic mouse models

**Figure 5:**
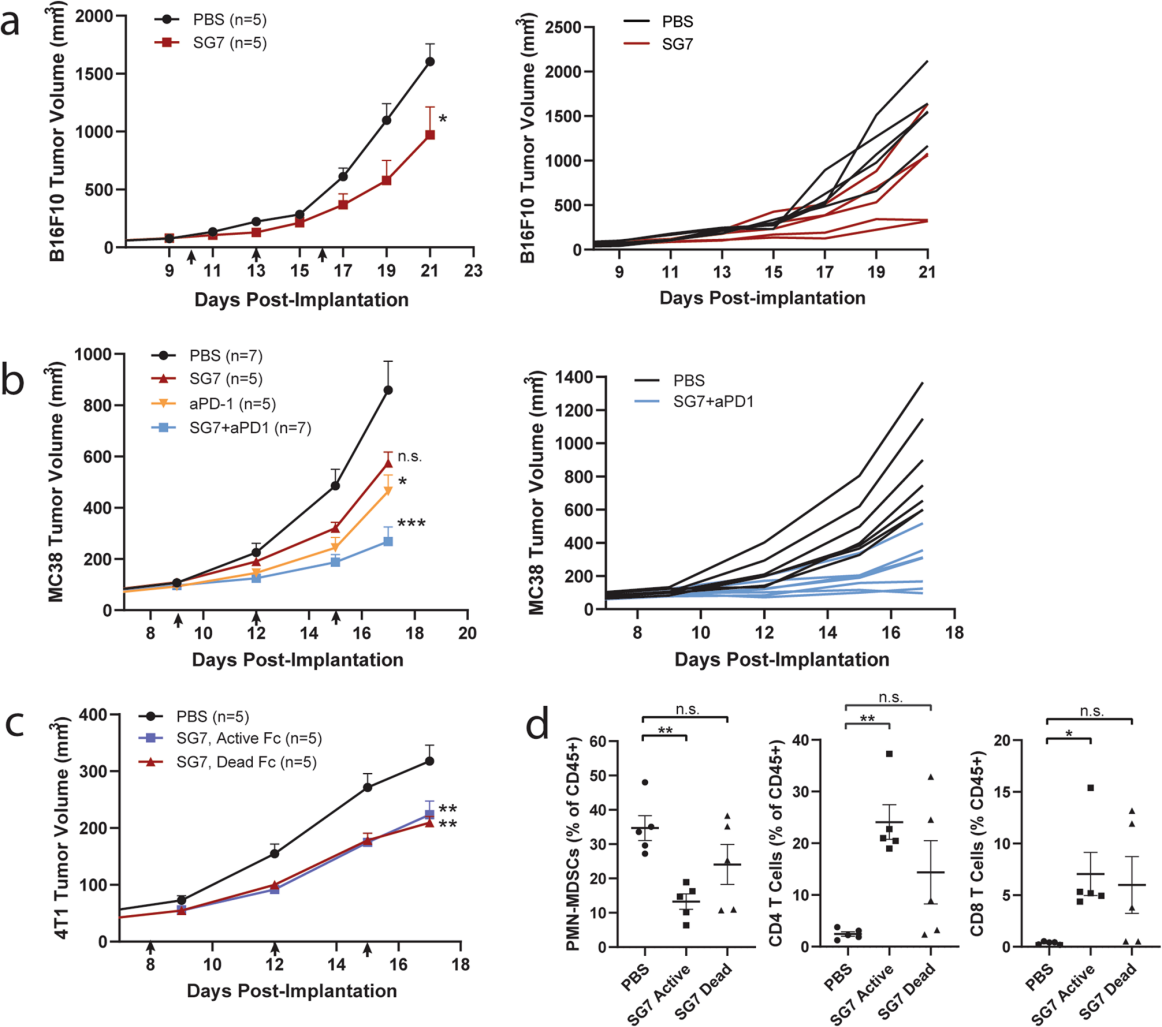
VISTA blockade with SG7 slows tumor growth in multiple syngeneic mouse models. (**a**) B16F10 tumor-bearing C57BL/6 mice were treated with 10 mg/kg SG7 (dead Fc; mIgG2a-LALA/PG) bi-weekly, starting on day 10 (red arrows). n = 5 mice per group; mean tumor volume of each group (left) and individual tumor growth curves (right). All mice were euthanized on Day 21 due to volume and ulceration of untreated tumors. These data are representative of two independent experiments. Mean + SEM are shown. (**b**) MC38 tumor-bearing C57BL/6 mice were treated with 30 mg/kg SG7 (dead Fc) and/or 5 mg/kg anti-PD1, starting on day 9 (black arrows); n = 7 mice per group; mean tumor volume of each group (left) and individual tumor growth curves (right); all mice were euthanized on Day 17 due to volume and ulceration of untreated tumors. These data are representative of two independent experiments. Mean + SEM are shown. (**c**) 4T1 tumor-bearing BALB/c mice were treated with 30 mg/kg SG7 (active Fc; mIgG2a) or 30 mg/kg SG7 (dead Fc) bi-weekly, starting on day 8 (black arrows). n = 5–7 mice per group as indicated. All mice were euthanized on Day 17 for tumor extraction. Mean + SEM are shown. P-values in **a**–**c** calculated by repeated measures two-way ANOVA (Tukey’s), asterisks denote significant difference from PBS (untreated), *p < 0.05, **p < 0.005, (**d**) Immune flow analysis of extracted 4T1 tumors on Day 17. The percentage of PMN-MDSCs, CD4 ^+^ T cells and CD8 ^+^ T cells out of total CD45^ +^ cells in each tumor sample are shown. Mean ± SEM are shown. P-values calculated by one-way ANOVA (DMCT), *p < 0.05, **p < 0.005.

Having shown that SG7 inhibited VISTA function and prevented PSGL-1 and VSIG3 binding to VISTA, we next tested the ability of SG7 to inhibit tumor growth in mouse models. While other researchers have demonstrated therapeutic efficacy of anti-VISTA antibodies using human VISTA knock-in mice^15,18^ or by using surrogate antibodies that only bind mouse VISTA^1–3,15^, the species cross-reactivity of SG7 enabled its testing in immunocompetent C57BL/6 and BALB/C mice. Two versions of SG7 antibody were expressed for in vivo testing; one with an ‘active Fc’ that is competent to engage FcγR-mediated effector functions (mIgG2a isotype) and one with a ‘dead Fc’ (LALA/PG mutations in the mIgG2a isotype) used to prevent depletion of healthy VISTA-expressing cells and allow the parsing of antibody-mediated VISTA blockade. First, we measured the efficacy of SG7 (dead Fc) administered biweekly for two weeks at 10 mg/kg in the aggressive B16F10 melanoma model and found SG7 significantly inhibited tumor growth compared to PBS-treated mice (Fig. 5a). The administration of PBS or a mIgG2a isotype control was shown to have no difference on tumor growth (Supplementary Fig. S7). Next, the effect of combining SG7 (dead Fc) and an anti-PD1 antibody was investigated in the MC38 colon carcinoma model. SG7 (dead Fc, 30 mg/kg) and anti-PD1 (5 mg/kg) were administered every 3–4 days for a total of three treatments each (Fig. 5b, black arrows). Mice in the combination treatment group had significantly smaller tumors than in the PBS-treated group, while both individual treatments were less effective (Fig. 5b). Finally, SG7 efficacy was evaluated in 4T1, a checkpoint inhibitor-resistant model of breast cancer. 4T1 tumors have been shown to have large numbers of MDSC infiltrates, particularly polymorphonuclear MDSCs (PMN-MDSCs)^19^. Because VISTA is highly expressed on PMN-MDSCs, we investigated the ability of SG7 to remodel the tumor microenvironment by depleting these inhibitory cells using the SG7 with an active Fc domain. 4T1-bearing mice were treated with 30 mg/kg SG7 antibody, containing either an active Fc or dead Fc, twice a week starting on Day 8 (black arrows) for a total of three injections. By day 17, both treatment groups demonstrated a minor, but significant decrease in tumor size compared to the PBS-treated group (Fig. 5c). At this time point, tumors were excised, dissociated into single cell suspensions, stained for T cell and myeloid cell markers, and analyzed by flow cytometry. The active Fc version of SG7 significantly and uniformly decreased the percentage of PMN-MDSCs and increased both CD4 ^+^ and CD8 ^+^ T cells (Fig. 5d). Other examined myeloid cell subsets such CD11b ^+^ macrophages, CD11c ^+^ dendritic cells, and M-MDSCs were unchanged among treatment groups (Supplementary Fig. S8). Although several tumors in the SG7 dead Fc treated group contained altered MDSC and T cell levels, these effects were not statistically significant. These results suggest that while SG7 can affect the levels of PMN-MDSCs and T cells in the tumor, blockade of VISTA rather than tumor remodeling likely contributes more strongly to the observed tumor growth effects.

## Discussion

Therapeutic development of antibodies often involves comprehensive characterization in small animal models before moving to toxicity studies in rodents and primates and clinical trials in humans. Biologics that are cross-reactive and functional in multiple species allows for experimental testing in animals with natural, autologous immune systems and the progression of the same molecule through the preclinical and clinical phases. While multiple groups are pursuing VISTA inhibitors, our work details a high affinity, species cross-reactive antibody against murine, cyno, and human VISTA. Another human/mouse cross-reactive antibody we are aware of, being developed by Hummingbird Biosciences, is reported to have significantly weaker affinity to mouse VISTA, which could cause disparate effects on safety and efficacy in mice versus humans. Other pre-clinical antibodies VSTB112 and BMS767, which bind only to human VISTA, have been characterized using human VISTA knock-in mice or mouse antibody surrogates. However, mice that express only human VISTA will not have natural VISTA/PSGL-1 or VISTA/VSIG3 interactions and VISTA blockade in this context will not show the full breadth of functionality or potential toxicity. Furthermore, availability of a mouse surrogate antibody allows for the use of immunocompetent mice, but does not support in vivo testing of the intrinsic qualities of the clinical antibody such as the effect of aggregation or formation of anti-drug antibodies. In addition, the surrogate antibody, by its nature, will not bind the exact same epitope as the clinical candidate, which can affect evaluation of activity or toxicity. Investigation of toxic side effects needs to be emphasized during the development process, particularly in light of early termination of Janssen’s clinical trial (NCT02671955) due to observed immune-related adverse events from their anti-human VISTA antibody.

The in vitro yeast surface display strategy used here for antibody discovery offers distinct advantages for generating an antibody like SG7, which binds with high affinity and species cross-reactivity to VISTA. While immunization of an animal with target antigen is a proven method for antibody generation, there is no selection pressure for generating binders to multiple species. Additionally, wild-type mice cannot be used due to the inherent selection against antibodies that target mouse antigens. With yeast display, antibodies can also be identified against cross-reactive epitopes that would otherwise not raise an immune response. The strategy outlined here of screening against one species (human antigen), mutagenesis of a biased library, and further screening against the another species (mouse antigen) allows for the expedient generation of fully human, high affinity, cross-reactive antibodies. The yeast-display workflow is broadly applicable to soluble antigen campaigns and could be used to de-risk pipelines through the development of species cross-reactive antibodies.

SG7 competes with VISTA binding to primary human and mouse T cells, and functions as a VISTA inhibitor. We observed VISTA-Fc binding to activated T cells at pH 6.0 but not at pH 7.4, reaffirming previous work detailing the pH selectivity of this interaction^15^. Additionally, we demonstrated the effectiveness of SG7 in restoring levels of T cell activation in an in vitro model using Jurkat-NFAT reporter T cells. This method using human Jurkat T cells and soluble VISTA allows for rapid screening of antibody functionality and can be broadly used by others developing VISTA inhibitors. We expected differences between individual antibody binding epitopes as SG7 binds mouse and human VISTA, while BMS767 and VSTB112 antibodies only bind human VISTA. In addition, BMS767 binds VISTA at pH 6.0 but not physiological pH^15^. Moreover, SG7 binds to hVISTA with a 25- to 50-fold tighter affinity than BMS767 or VSTB112 (Supplementary Fig. S9), which might translate into increased therapeutic efficacy. SG7 binding to VISTA relied on H122 and E125, two residues in the histidine-rich tip of VISTA, and was also heavily influenced, directly or indirectly, by residues F36 and K38. In comparison, BMS767 binding to VISTA is dependent on histidine residues in the tip (H121, H122) as well as a number of residues in the front C–C’ loop side of the protein. The importance of these histidines, as well as H123 and H68, which were not identified by the SG7 yeast-display screen, could potentially explain why BMS767 has pH-dependent binding to VISTA but SG7 does not (Supplementary Fig.S9). VSTB112 appeared to bind many of the same VISTA residues as BMS767 (Y37, R54, F62, Q63) but was not affected as significantly by mutation of histidines in the tip (H121, H122), which may also explain why binding of VSTB112 to VISTA is not pH-dependent (Supplementary Fig.S9). SG7 and VSTB112 share H122 as an involved binding residue, potentially explaining why these antibodies cannot bind to VISTA simultaneously.

The field has long sought to identify and characterize the binding partner for VISTA. A recent study identified a pH-dependent binding interaction between VISTA and PSGL-1^15^ and two independent protein interaction screens identified VSIG3 (also called IGSF11) as a VISTA binding partner^13,14^. Providing confirmation for both these findings, we demonstrated that VISTA binds both PSGL-1 and VSIG3. The PSGL-1 binding interaction was heavily dependent on low pH, while the weaker affinity VSIG3 interaction bound slightly better at physiological pH. Our confirmation of two distinct binding interactions opens the possibility that VISTA has two separate roles: (1) a receptor on T cells that is ligated by VSIG3 and (2) a ligand on myeloid cells that binds to PSGL-1 receptor on T cells. The SG7 antibody, as well as BMS767 and VSTB112, blocked both PSGL-1 and VSIG3 interactions, a feature which may end up being important for effective VISTA inhibition once the roles of these proteins are better understood. Through recombinant expression of a panel of VISTA mutants, we also propose regions on the VISTA protein that are important for PSGL-1 and VSIG3 binding. Interestingly, the predicted epitopes of both proteins have significant overlap, especially with residues in the C–C’ loop and adjacent helix (R54, Q63) and with residues in beta strand C (F36, K38). The dependence of PSGL-1 binding on multiple histidines (H121, H122) could play a role to its pH selectivity compared to VSIG3, although other residues likely contribute as mutations in these two residues are not sufficient to confer pH sensitivity with SG7. All three antibodies and both PSGL-1 and VSIG3 proteins appear to utilize H122 in VISTA binding, albeit to different extents, which could explain why SG7, BMS767, and VSTB112 can all effectively block both PSGL-1 and VSIG3 interactions. Analysis of our panel of VISTA mutants also revealed that the E125A and F62A mutations improve binding to PSGL-1 and VSIG3, respectively. Agonism of the VISTA pathway has been shown to decrease inflammation^2,20^ and a higher affinity VISTA molecule could therefore have applications in autoimmune disease.

While other groups have demonstrated the efficacy of combining VISTA and PD-1/PD-L1 blockade^15,21^, our work details a species cross-reactive VISTA antagonist that might therefore more directly translate into a clinical drug. In B16F10 and 4T1 models, a version of SG7 that is deficient in engaging FcyR-mediated effector functions showed significant efficacy compared to PBS-treated mice, suggesting that blocking VISTA binding, rather than depletion of VISTA-expressing cells, is the primary mechanism of action. Treatment with such a non-depleting antibody could prevent toxicity from clearance of healthy VISTA-expressing cells and potentially avoid immune adverse events, which may have been problematic in a recent clinical trial with VSTB112. Future work investigating the effect of VISTA and PD-1 combination blockade on other co-inhibitory receptors (TIM-3, LAG-3, TIGIT) and inhibitory cell types (regulatory T cells, tumor associated macrophages) will be necessary to more comprehensively define the therapy’s mechanism of action. The data presented here detail the engineering and functional characterization of an intriguing cross-reactive VISTA antibody and provides insight into the binding epitopes and interactions of VISTA to help guide future therapeutic development.

## Methods

### Screening a yeast-displayed scFv library for VISTA Binders

Yeast of the S. *cerevisiae* EBY100 strain were prepared for yeast-surface display by transformation of scFv genes as described previously^22,23^. The xEmplar library consists of ~ 10^9^ scFv mutants fused to the yeast cell-wall protein Aga2p. Yeast were grown on a shaking platform set to 235 RPM in minimal media with dextrose (SD-CAA) at 30 °C, and induced for surface expression in minimal media with galactose (SG-CAA) at 20 °C. The first screen against human VISTA was performed with magnetic-activated cell sorting (MACS), as described previously^23^. Briefly, VISTA antigen was biotinylated using EZ-Link NHS-Ester (Thermo Fisher Scientific, A39256) according to the manufacture’s protocol. Biotinylated antigen bound to Dynabeads M-280 Streptavidin (Thermo Fisher Scientific, 11205D) was used for positive selection and unloaded beads were used for negative selection. Yeast pools that did not bind to negative selection beads but did bind to positive selection beads were grown for subsequent rounds of fluorescence-activated cell sorting (FACS). Equilibrium sorts were performed in which yeast were incubated at 4 °C for 4–6 h in phosphate-buffered saline (PBS) containing 1 mg/mL BSA (PBS/BSA) with the following concentrations of hVISTA-Fc (Sort 1.2, 500 nM; Sort 1.3, 50 nM; and sort 1.4, 5 nM). A 1:5,000 dilution of chicken anti-c-Myc (Invitrogen, A21281) was added with antigen in each sort round. Yeast were then washed and labeled on ice with 1:250 dilution of secondary antibodies for binding (Streptavidin 488, ThermoFisher S11223 or anti-human 647, ThermoFisher A21445) and expression (anti-chicken 647, Abcam ab150171 or anti-chicken 488, ThermoFisher A11039). Yeast after sort 1.4 were sequenced and analyzed for binding to hVISTA-Fc or mVISTA-His (see *Binding analysis of scFv clones displayed on yeast*). A new library of scFv mutants was created (see *Creation of biased scFv library for affinity maturation*) and equilibrium sort rounds were performed as before against the following concentrations of antigen (Sort 2.1, 100 nM mVISTA-His and Sort 2.2, 50 nM mVISTA-His) A final off-rate sort was performed in Sort 2.3 by incubating yeast pools with 10 nM hVISTA-His, washing cells with PBS + 1 mg/mL BSA, and incubating with 1 µM non-biotinylated hVISTA-His as a competitor. The resulting yeast pool was sequenced and analyzed for high affinity single clones (see *Single clone binding analysis*).

### Creation of biased scFv library for affinity maturation

A DNA mixture composed of a weight ratio of 75% pooled sort 1.4 and 25% clone V9 was subjected to error-prone PCR. Mutations were introduced across the whole gene using low-fidelity Taq polymerase (Invitrogen) and nucleotide analogs 8-oxo-dGTP and dPTP (TriLink Biotech) as described previously^23,24^. The dNTP analog concentration was optimized to achieve an average of 2–3 amino acids per gene. This library was amplified and purified using gel electrophoresis. Empty yeast displayed vector was cut using NheI and BamHI restriction sites. The amplified insert and cut vector were electroporated in a 4:1 DNA weight ratio into yeast engineered for surface protein display^22,25^, where they were assembled in vivo through homologous recombination. Library size was determined to be ~ 2 × 10^8^ by dilution plating. The new library was affinity matured against mouse VISTA (see *Screening a yeast-displayed scFv library for VISTA binders*).

### Recombinant protein expression

#### VISTA, VSIG3, PSGL-1 Proteins

Recombinant human and mouse VISTA proteins were expressed recombinantly for sorting and binding experiments. Three wild-type versions of each protein were made from the gene sequence of the extracellular domain linked to: (1) a 6x-His-tag, (2) an Fc domain (IgG1 for human VISTA or mIgG2a for mouse VISTA), or (3) a LALA/PG mutated version of Fc domain to eliminate Fc receptor interactions in T cell binding assays^26^. For His-tagged proteins, a hexahistidine epitope was included on the C-terminus of human VISTA ECD (Met1-Ala194, UniProt #Q9H7M9) or mouse VISTA ECD (Met1-Ala191, Uniprot #Q9D659). For Fc-fusion proteins, human VISTA ECD was followed by a GGGGS linker and then hIgG1 Fc (Glu99-Lys330, Uniprot #P01857) while mouse VISTA ECD was followed by a GGGGS linker and then the mIgG2a Fc (Glu98-Lys330, Uniprot #P01863). The LALA/PG mutations of L117A, L118A, and P212G were introduced into the backbone of hIgG1 and mIgG2a. The DNA encoding each of the six proteins were ordered as a gblock Gene Fragment (IDT) and cloned into the cytomegalovirus-driven adenoviral shuttle vector pAdd2 using standard Gibson cloning at EcoRI/XhoI vector cut sites. Protein was expressed using the Expi293 system (Thermo Fisher Scientific, A14526) according to manufacturer’s protocol and purified from culture supernatant using nickel affinity chromatography for His-tagged proteins or protein A affinity chromatography for Fc fusion proteins.

Soluble hVISTA-Fc mutants were expressed for epitope analysis with VSIG3/PSGL-1. The ECD for each mutant was ordered as gene fragments (Twist Biosciences) and joined with the Fc domain of mIgG2a (Glu98-Lys330, Uniprot #P01863) using overlap extension PCR. Fragments were joined with mIgG2a Fc to allow the use of anti-mouse HRP antibodies for detection in binding ELISAs against human IgG1 Fc-tagged proteins. The full VISTA-Fc genes were cloned into the pAdd2 vector using standard Gibson cloning, as described above. The ECD of VSIG3 (Leu23-Gly241, Uniprot #Q5DX21) was ordered as a gblock gene fragment (IDT), joined with the Fc domain of hIgG1 (Glu99-Lys330, Uniprot #P01857), and cloned into pAdd2 using standard Gibson cloning. DNA encoding hVISTA-Fc mutants and hVSIG3-Fc were transfected into Expi293 cells and expressed according to manufacturer’s protocol. Protein was purified from supernatant using protein A affinity chromatography. Human PSGL-1-Fc was purchased from R&D Systems (3345-PS).

#### Antibodies

SG7 was expressed as a full-length mIgG2a antibody or as a LALA/PG mutated version to eliminate Fc receptor binding^26^. VSTB112 and BMS767 were expressed as full human IgG1 antibodies. Sequences are listed in Table 1. Full heavy chain and light chain sequences for all three antibodies were ordered as gblock gene fragments (IDT) and individually cloned into pAdd2 using standard Gibson cloning, as described above. Antibody genes were transfected into Expi293 cells (Thermo Fisher Scientific, A14526) in a 1:1 weight ratio of heavy chain to light chain and expressed according to manufacturer’s protocol. Antibody was purified from the supernatant using protein A affinity chromatography.

**Table 1:** Sequence list.

SG7 HC	*MGWSLILLFLVAVATGVHS*QVQLVQSGAEVKKPGSSVKVSCKASGGIFSSYAISWVRQAPGQGLEWMGGIIPIFGTANYAQKFQGRVTITADESTSTAYMELSSLRSEDTAVYYCARPVRSGPDYLQHWGQGTLVTVSSAKTTAPSVYPLAPVCGGTTGSSVTLGCLVKGYFPEPVTLTWNSGSLSSGVHTFPALLQSGLYTLSSSVTVTSNTWPSQTITCNVAHPASSTKVDKKIEPRVPITQNPCPPLKECPPCAAPDLLGGPSVFIFPPKIKDVLMISLSPMVTCVVVDVSEDDPDVQISWFVNNVEVHTAQTQTHREDYNSTLRVVSALPIQHQDWMSGKEFKCKVNNRALPSPIEKTISKPRGPVRAPQVYVLPPPAEEMTKKEFSLTCMITGFLPAEIAVDWTSNGRTEQNYKNTATVLDSDGSYFMYSKLRVQKSTWERGSLFACSVVHEGLHNHLTTKTISRSLGK
SG7 HC—LALA/PG	*MGWSLILLFLVAVATGVHS*QVQLVQSGAEVKKPGSSVKVSCKASGGIFSSYAISWVRQAPGQGLEWMGGIIPIFGTANYAQKFQGRVTITADESTSTAYMELSSLRSEDTAVYYCARPVRSGPDYLQHWGQGTLVTVSSAKTTAPSVYPLAPVCGGTTGSSVTLGCLVKGYFPEPVTLTWNSGSLSSGVHTFPALLQSGLYTLSSSVTVTSNTWPSQTITCNVAHPASSTKVDKKIEPRVPITQNPCPPLKECPPCAAPD**AA**GGPSVFIFPPKIKDVLMISLSPMVTCVVVDVSEDDPDVQISWFVNNVEVHTAQTQTHREDYNSTLRVVSALPIQHQDWMSGKEFKCKVNNRAL**G**SPIEKTISKPRGPVRAPQVYVLPPPAEEMTKKEFSLTCMITGFLPAEIAVDWTSNGRTEQNYKNTATVLDSDGSYFMYSKLRVQKSTWERGSLFACSVVHEGLHNHLTTKTISRSLGK
SG7 LC	*MDMRVPAQLLGLLLLWLPGARC*DIQMTQSPSTLSASVGDRVTITCRASQSISSWLAWYQQKPGKAPKLLIYDASSLESGVPSRFSGSGSGTEFTLTISSLQPDDFATYYCQQYNSYSLTFGGGTKVEIKRADAAPTVSIFPPSSEQLTSGGASVVCFLNNFYPKDINVKWKIDGSERQNGVLNSWTDQDSKDSTYSMSSTLTLTKDEYERHNSYTCEATHKTSTSPIVKSFNRNEC
human VISTA ECD Uniprot	*MGVPTALEAGSWRWGSLLFALFLAASLGPVAA*FKVATPYSLYVCPEGQNVTLTCRLLGPVDKGHDVTFYKTWYRSSRGEVQTCSERRPIRNLTFQDLHLHHGGHQAANTSHDLAQRHGLESASDHHGNFSITMRNLTLLDSGLYCCLVVEIRHHHSEHRVHGAMELQVQTGKDAPSNCVVYPSSSQDSENITAA
cyno VISTA ECD Uniprot	*MGVPTAPEAGCWRWGSLLFALFLAASLGPVAA*FKVATLYSLYVCPEGQNVTLTCRVFGPVDKGHDVTFYKTWYRSSRGEVQTCSERRPIRNLTFQDLHLHHGGHQAANTSHDLAQRHGLESASDHHGNFSITMRNLTLLDSGLYCCLVVEIRHHHSEHRVHGAMELQVQTGKDAPSSCVAYPSSSQESENITAA
mouse VISTA ECD Uniprot	*MGVPAVPEASSPRWGTLLLAIFLAASRGLVAA*FKVTTPYSLYVCPEGQNATLTCRILGPVSKGHDVTIYKTWYLSSRGEVQMCKEHRPIRNFTLQHLQHHGSHLKANASHDQPQKHGLELASDHHGNFSITLRNVTPRDSGLYCCLVIELKNHHPEQRFYGSMELQVQAGKGSGSTCMASNEQDSDSITAA
mVISTA-HIS	*MGVPAVPEASSPRWGTLLLAIFLAASRGLVAA*FKVATPYSLYVCPEGQNVTLTCRLLGPVDKGHDVTFYKTWYRSSRGEVQTCSERRPIRNLTFQDLHLHHGGHQAANTSHDLAQRHGLESASDHHGNFSITMRNLTLLDSGLYCCLVVEIRHHHSEHRVHGAMELQVQTGKDAPSNCVVYPSSSQDSENITAAHHHHHH
hVISTA-HIS	*MGVPTALEAGSWRWGSLLFALFLAASLGPVAA*FKVATPYSLYVCPEGQNVTLTCRLLGPVDKGHDVTFYKTWYRSSRGEVQTCSERRPIRNLTFQDLHLHHGGHQAANTSHDLAQRHGLESASDHHGNFSITMRNLTLLDSGLYCCLVVEIRHHHSEHRVHGAMELQVQTGKDAPSNCVVYPSSSQDSENITAAHHHHHH
hVISTA-Fc	*MGVPTALEAGSWRWGSLLFALFLAASLGPVAA*FKVATPYSLYVCPEGQNVTLTCRLLGPVDKGHDVTFYKTWYRSSRGEVQTCSERRPIRNLTFQDLHLHHGGHQAANTSHDLAQRHGLESASDHHGNFSITMRNLTLLDSGLYCCLVVEIRHHHSEHRVHGAMELQVQTGKDAPSNCVVYPSSSQDSENITAAGSGGGGSEPKSCDKTHTCPPCPAPELLGGPSVFLFPPKPKDTLMISRTPEVTCVVVDVSHEDPEVKFNWYVDGVEVHNAKTKPREEQYNSTYRVVSVLTVLHQDWLNGKEYKCKVSNKALPAPIEKTISKAKGQPREPQVYTLPPSRDELTKNQVSLTCLVKGFYPSDIAVEWESNGQPENNYKTTPPVLDSDGSFFLYSKLTVDKSRWQQGNVFSCSVMHEALHNHYTQKSLSLSPGK
mVISTA-Fc	*MGVPAVPEASSPRWGTLLLAIFLAASRGLVAA*FKVTTPYSLYVCPEGQNATLTCRILGPVSKGHDVTIYKTWYLSSRGEVQMCKEHRPIRNFTLQHLQHHGSHLKANASHDQPQKHGLELASDHHGNFSITLRNVTPRDSGLYCCLVIELKNHHPEQRFYGSMELQVQAGKGSGSTCMASNEQDSDSITAAGSGGGGSEPRGPTIKPCPPCKCPAPNLLGGPSVFIFPPKIKDVLMISLSPIVTCVVVDVSEDDPDVQISWFVNNVEVHTAQTQTHREDYNSTLRVVSALPIQHQDWMSGKEFKCKVNNKDLPAPIERTISKPKGSVRAPQVYVLPPPEEEMTKKQVTLTCMVTDFMPEDIYVEWTNNGKTELNYKNTEPVLDSDGSYFMYSKLRVEKKNWVERNSYSCSVVHEGLHNHHTTKSFSRTPGK
hVISTA-Fc—LALA/PG	*MGVPTALEAGSWRWGSLLFALFLAASLGPVAA*FKVATPYSLYVCPEGQNVTLTCRLLGPVDKGHDVTFYKTWYRSSRGEVQTCSERRPIRNLTFQDLHLHHGGHQAANTSHDLAQRHGLESASDHHGNFSITMRNLTLLDSGLYCCLVVEIRHHHSEHRVHGAMELQVQTGKDAPSNCVVYPSSSQDSENITAAGSGGGGSEPKSCDKTHTCPPCPAPE**AA**GGPSVFLFPPKPKDTLMISRTPEVTCVVVDVSHEDPEVKFNWYVDGVEVHNAKTKPREEQYNSTYRVVSVLTVLHQDWLNGKEYKCKVSNKALPA**G**IEKTISKAKGQPREPQVYTLPPSRDELTKNQVSLTCLVKGFYPSDIAVEWESNGQPENNYKTTPPVLDSDGSFFLYSKLTVDKSRWQQGNVFSCSVMHEALHNHYTQKSLSLSPGK
mVISTA-Fc—LALA/PG	*MGVPAVPEASSPRWGTLLLAIFLAASRGLVAA*FKVTTPYSLYVCPEGQNATLTCRILGPVSKGHDVTIYKTWYLSSRGEVQMCKEHRPIRNFTLQHLQHHGSHLKANASHDQPQKHGLELASDHHGNFSITLRNVTPRDSGLYCCLVIELKNHHPEQRFYGSMELQVQAGKGSGSTCMASNEQDSDSITAAGSGGGGSEPRGPTIKPCPPCKCPAPN**AA**GGPSVFIFPPKIKDVLMISLSPIVTCVVVDVSEDDPDVQISWFVNNVEVHTAQTQTHREDYNSTLRVVSALPIQHQDWMSGKEFKCKVNNKDL**G**APIERTISKPKGSVRAPQVYVLPPPEEEMTKKQVTLTCMVTDFMPEDIYVEWTNNGKTELNYKNTEPVLDSDGSYFMYSKLRVEKKNWVERNSYSCSVVHEGLHNHHTTKSFSRTPGK
VSTB112 HC	*MGWSLILLFLVAVATGVHS*QVQLVQSGAEVKKPGSSVKVSCKASGGTFSSYAISWVRQAPGQGLEWMGGIIPIFGTANYAQKFQGRVTITADESTSTAYMELSSLRSEDTAVYYCARSSYGWSYEFDYWGQGTLVTVSSASTKGPSVFPLAPSSKSTSGGTAALGCLVKDYFPEPVTVSWNSGALTSGVHTFPAVLQSSGLYSLSSVVTVPSSSLGTQTYICNVNHKPSNTKVDKKVEPKSCDKTHTCPPCPAPELLGGPSVFLFPPKPKDTLMISRTPEVTCVVVDVSHEDPEVKFNWYVDGVEVHNAKTKPREEQYNSTYRVVSVLTVLHQDWLNGKEYKCKVSNKALPAPIEKTISKAKGQPREPQVYTLPPSRDELTKQVSLTCLVKGFYPSDIAVEWESNGQPENNYKTTPPVLDSDGSFFLYSKLTVDKSRWQQGNVFSCVMHEALHNHYTQKSLSLSPGK
VSTB112 LC	*MDMRVPAQLLGLLLLWLPGARC*DIQMTQSPSSLSASVGDRVTITCRASQSIDTRLNWYQQKPGKAPKLLIYSASSLQSGVPSRFSGSGSGTDFTLTISSLQPEDFATYYCQQSAYNPITFGQGTKVEIKRTVAAPSVFIFPPSDEQLKSGTASVVCLLNNFYPREAKVQWKVDNALQSGNSQESVTEQDSDKSTYSLSSTLTSKADYEKHKVYACEVTHQGLSSPVTKSFNRGEC
BMS767 HC	*MGWSLILLFLVAVATGVHS*EVQLVESGGGLVQPGKSLRLSCAASGFTLEDYAMHWVRQAPGKGLEWVSGIDWNSENIGYADSVKGRFTISRDNAKNSLYLQMNSLRTEDTALYYCAKVPGYSGGWIDAEDDWGQGTMVTVSSASTKGPSVFPLAPSSKSTSGGTAALGCLVKDYFPEPVTVSWNSGALTSGVHTFPAVLQSSGLYSLSSWTVPSSSLGTQTYICNVNHKPSNTKVDKRVEPKSCDKTHTCPPCPAPEAEGAPSVFLFPPKPKDTLMISRTPEVTCVWDVSHEDPEVKFNWYVDGVEVHNAKTKPREEQYNSTYRWSVLTVLHQDWLNGKEYKCKVSNKALPAPIEKTISKAKGQPREPQVYTLPPSREEMTKNQVSLTCLVKGFYPSDIAVEWESNGQPENNYKTTPPVLDSDGSFFLYSKLTVDKSRWQQGNVFSCSVMHEALHNHYTQKSLSLSPG
BMS767 LC	*MDMRVPAQLLGLLLLWLPGARC*EIVLTQSPGTLSLSPGERATLSCRASQSVSSSYLAWYQQKPGQAPRLLIYGASSRATGIPDRFSGSGSGTDFTLTISRLEPEDFAVYYCQQYGSSPFTFGPGTKVDIKRTVAAPSVFIFPPSDEQLKSGTASWCLLNNFYPREAKVQWKVDNALQSGNSQESVTEQDSKDSTYSLSSTLTLSKADYEKHKVYACEVTHQGLSSPVTKSFNRGEC
Clone V9.7	DIQMTQSPSTLSASVGDRVTITCRASQSISSWLAWYQQKPGKAPKLLIYDASSLESGVPSRFSGSGSGTEFTLTISSLQPDDFATYYCQQYNSYSLTFGGGTKVEIKGTTAASGSSGGSSSGAQVQLVQSGAEVKKPGSSVKVSCKASGGIFSSYAISWVRQAPGQGLEWMGGIIPIFGTANYAQKFQGRVTITADESTSTAYMELSSLRSEDTAVYYCARPVRSGPDYLQHWGQGTLVTVSS

### Binding analysis of scFv clones displayed on yeast

Yeast pools after sort 1.4 and 2.3 were grown in minimal media (SD-CAA) at 30 °C, shaking at 235 RPM. After overnight growth, plasmid DNA was extracted from yeast using a Zymoprep kit (Zymo Research Corp) and transformed into DH10b electrocompetent *E. coli*. Fifty colonies were picked and sequenced by MCLAB (Molecular Cloning Laboratories). Sequences were analyzed with Geneious software and unique clones were identified (18 from Round 1, 2 from Round 2). DNA from each unique clone was transformed back into yeast using the Frozen-EZ Yeast Transformation II Kit (Zymo Research, #T2001) and plated on minimal media (SD-CAA) plates. After three days of growth at 30 °C, colonies for each individual clone were picked and grown in minimal media (SD-CAA) at 30 °C overnight and then induced for surface expression in minimal media with galactose (SG-CAA) at 20 °C overnight. Clones after round 1 of sorting (post sort 1.4) were incubated with 10 nM hVISTA-Fc or 100 nM mVISTA-His while clones after round 2 of sorting (post sort 2.3) were incubated with a titration of hVISTA-Fc or mVISTA-His to obtain full binding curves. Binding reactions were incubated at 4 °C for 12 h to allow interactions to reach equilibrium. Yeast were labeled with the same reagents using protocols as described for library screening and analyzed by flow cytometry on a BD Accuri. The binding signal was normalized for expression using FlowJo software and analyzed using Binding – saturation (one site) non-linear regression on GraphPad Prism. Binding of Clone V9.7 (scFv fragment of SG7) to mouse, cyno, and human VISTA was measured on yeast to demonstrate species cross-reactivity (Supplementary Fig. S2). Yeast-displayed clone 9.7 was incubated with titrations of mVISTA-Fc, cyno VISTA-Fc (R&D Systems, Cat# 9408-B7), or hVISTA-Fc in PBS/BSA (pH 7.4) at 4 °C for 12 h to allow interactions to reach equilibrium. Binding signal was measured on a BD Accuri and analyzed on FlowJo and GraphPad Prism as before.

### Binding analysis of VISTA displayed on yeast

Binding of yeast-displayed hVISTA (Phe33-Ala194, Uniprot #Q9H7M9) to soluble SG7, BMS767, and VSTB112 antibodies (Supplementary Fig. S5A, S5B) was measured. Titrations of antibody were incubated with yeast in PBS/BSA (pH 6.0 or pH 7.4) at 4 °C for 12 h to allow interactions to reach equilibrium. Binding signal was measured on a BD Accuri and analyzed on FlowJo and GraphPad Prism as before. PBS/BSA at pH 6.0 was prepared by titrating in hydrochloric acid to standard PBS + 1 mg/mL BSA. hVISTA and mVISTA mutants were displayed on yeast for single clone analysis (Fig. 3c,e). Mutants based on wild-type hVISTA ECD (Phe33-Ala194, Uniprot #Q9H7M9) or wild-type mVISTA ECD (Phe33-Ala191, Uniprot # Q9D659) were induced for surface expression on yeast as described previously. Yeast-displayed clones were incubated with a concentration of each antibody at its estimated K_d_ value (SG7: 300 pM; BMS: 3 nM; or VSTB: 3 nM) in PBS/BSA at pH 7.4 (SG7, VSTB112) or pH 6.0 (BMS767) at 4 °C for 12 h to allow interactions to reach equilibrium. Binding signal was measured on a BD Accuri and analyzed on FlowJo and GraphPad Prism as before.

### Measuring protein affinity using the kinetic exclusion assay (KinExA)

A KinExA 3200 instrument (Sapidyne Instruments) was used to measure SG7 antibody affinity to mVISTA-His and hVISTA-His under equilibrium conditions. Polymethyl methacrylate beads (98 micron, PMMA beads; Sapidyne Instruments, Cat# 440107) coated with SG7 was used to detect free hVISTA-His or mVISTA-His. Adsorption coating of 200 mg of beads was performed using 40 µg of SG7 for 2 h at room temperature. The protein solution was aspirated and beads were blocked using PBS with 10 mg/mL BSA for 2 h at room temperature. Beads were stored in blocking buffer and used within 1 week. Soluble hVISTA-His or mVISTA-His was incubated at a constant concentration with serially diluted SG7 in KinExA Running Buffer (PBS + 10 mg/mL BSA + 0.02% sodium azide). For hVISTA, SG7 was varied from 1.69 pM to 100 nM and hVISTA-His was held at 250 pM. For mVISTA, SG7 was varied from 5.08 pM to 300 nM and mVISTA-His was held at 1 nM. Proteins were incubated for 18 h before being run on the KinExA instrument as an Equilibrium Assay. Binding of free hVISTA or mVISTA was detected with anti-6 × HIS Dylight 649 Antibody (Rockland Immunochemicals Inc, Cat# 200-343-382). Each sample was measured twice, and the data were globally analyzed using n-curve analysis with KinExA Pro 3.6.2 software (Sapidyne Instruments) to obtain the K_d_ value.

### Enzyme-linked immunosorbent assays (ELISAs)

Four different ELISAs were performed: (1) Sandwich ELISA with SG7 and competitor antibodies (Fig. 2d), (2) PSGL-1-Fc or VSIG3-Fc direct ELISAs with soluble WT hVISTA-Fc for apparent K_d_ measurement (Fig. 3a), (3) PSGL-1-Fc or VSIG3-Fc competition ELISAs with pre-complexed hVISTA-Fc (Fig. 3b,c), (4) PSGL-1-Fc or VSIG3-Fc direct ELISAs with soluble hVISTA-Fc mutants for epitope mapping (Fig. 3d). In all cases, clear, flat-bottom 96-well plates (Thermo Fisher Scientific, #12–565-136) were incubated with 50 µL target protein in PBS at 4 °C overnight, blocked with 400 µL PBS + 50 mg/mL BSA at room temperature for 2 h, washed three times between each step with 400 µL PBS + 0.1% Tween-20, bound with reagents diluted in 50 µL PBS + 1 mg/mL BSA + 0.1% Tween-20, and detected on a microplate reader (Synergy H4, BioTek) after 15 min incubation with substrate solution (1-Step Ultra TMB, Thermo Fisher, 34028) and addition of 2 M sulfuric acid to stop the reaction.

*For ELISA #1*, BMS767, VSTB112, a hIgG1 isotype control Ab (−), and an anti-His antibody (+) were coated at 5 µg/mL. 1 nM hVISTA-His was added to all wells and incubated at room temperature for 2 h. SG7 was serially diluted and then added to VISTA-competitor antibody complexes at 4 °C for 20 min. A 1:7,500 dilution of anti-mouse HRP (Fisher Scientific, Cat# 62-6520) was used for detection of bound SG7. *For ELISA #2*, 5 µg/mL of PSGL-1 or VSIG3-Fc was coated and a serial dilution of hVISTA-Fc was incubated on pre-coated wells for 2 h at 4 °C. A 1:7,500 dilution of anti-human HRP (Abcam, ab97175) was used for detection of bound hVISTA-Fc. Individual values were fit to a binding curve using non-linear regression on GraphPad Prism (binding-one site) for apparent K_d_ calculations. *For ELISA #3*, 5 µg/mL of PSGL-1-Fc or 10 µg/mL VSIG3-Fc was coated at pH 7.4. All following steps were conducted at pH 6. Additionally, hVISTA-Fc was biotinylated with EZ-Link NHS Ester (Thermo Fisher Scientific, A39256) according to the manufacturer’s protocol. A serial dilution of SG7, BMS767, VSTB112, or an isotype control (SG7 scrambled antibody) was pre-complexed with 100 nM hVISTA-Fc-Biotin for 2 h at room temperature. Complexed protein was then incubated with pre-coated wells for 2 h at room temperature. A 1:7,500 dilution of Streptavidin-HRP (Abcam, ab7403) was used for detection of bound hVISTA-Fc. *For ELISA #4*, 5 µg/mL of PSGL-1-Fc or VSIG3-Fc was coated and 11 different hVISTA-Fc mutants were added near the approximate K_d_ concentration of each interaction (5 nM for hVISTA/PSGL-1 and 150 nM for hVISTA/VSIG3). Binding reactions were incubated for 2 h at room temperature and then a 1:7,500 dilution of anti-mouse HRP was used to detect bound hVISTA-Fc mutants.

### Primary T cell binding assays

Primary mouse T cells were obtained by sacrificing a C57BL/6 mouse and extracting its spleen. The spleen was minced and pushed through a 70 µM cell strainer (Fisher Scientific, 08-771-2). Red blood cells were lysed with ACK Lysis Buffer (Fisher Scientific, A1049201) and splenocytes were pelleted and washed with complete RPMI media: RPMI (Gibco, 11875119) supplemented with 10% fetal bovine serum (Thermo Fisher Scientific, 26-140-079), 1% penicillin–streptomycin (Fisher Scientific, 15-140-122), and 0.05 mM 2-mercaptoethanol (Sigma, M6250). To activate splenocytes and enrich for T cells, splenocytes were cultured in a 96-well tissue culture dish (Greiner, 655160) with 30 U/mL recombinant human IL-2 (BioLegend, 589102) and CD3/CD28 Dynabeads (Fisher Scientific, 11456D) according to the manufacturer’s protocol. After 72 h at 37 °C, cells were removed from the plate by washing out wells with PBS + 0.1% BSA (pH 6.0). 100 nM mouse VISTA-Fc-Biotin was incubated with a serial dilution of SG7 for 1 h at room temperature in PBS + 0.1% BSA, pH 6.0. For a negative control, a serial dilution of VSTB112 was pre-complexed with mVISTA-Fc-Biotin. Cells were added to pre-complexed SG7/VISTA and incubated for 1 h at 4 °C. Cells were washed twice with PBS + 0.1% BSA (pH 6.0) and bound mouse VISTA was detected with streptavidin-488 (ThermoFisher, S11223). Binding signal was measured on a BD Accuri flow cytometer and competition binding curves were fit with nonlinear regression ([Inhibitor] vs response) with Graphpad Prism.

Frozen primary human T cells (CD4 + CD8) were obtained from the Mackall group at Stanford Medicine. Briefly, T cells were isolated from human buffy coats by negative selection using the RosetteSep Human T Cell Enrichment Cocktail kit (STEMCELL Technologies). Cells were thawed into complete RPMI Media and proliferated in a T-25 flask (E&K, EK-49175) with 100 U/mL recombinant human IL-2 (BioLegend, 589102) and human activator CD3/CD28 Dynabeads (Fisher Scientific, 11161D), according to the manufacturer’s protocol. After 72 h at 37 °C, cells were removed from the flask, pelleted, and washed with PBS + 0.1% BSA (pH 6.0). 100 nM human VISTA-Fc-Biotin was incubated with a serial dilution of SG7 for 1 h at room temperature in PBS + 0.1% BSA (pH 6.0). For a negative control, a serial dilution of an isotype control antibody (SG7-scrambled) was pre-complexed with mVISTA-Fc-Biotin. Cells were added to pre-complexed SG7/VISTA and incubated for 1 h at 4 °C. Cells were washed twice with PBS + 0.1% BSA and bound human VISTA was detected with streptavidin-488 (ThermoFisher, S11223). Binding signal was measured on a BD Accuri flow cytometer and competition binding curves were fit with nonlinear regression ([Inhibitor] vs response) with Graphpad Prism.

### Jurkat T cell activation assay

Jurkat T cells with an NFAT reporter element linked to expression of blue fluorescent protein (BFP) was obtained from the Qi group at Stanford Bioengineering. Jurkat T cells were cultured in RPMI (Gibco, 11875119) supplemented with 10% fetal bovine serum (Thermo Fisher Scientific, 26-140-079) and 1% penicillin–streptomycin (Fisher Scientific, 15-140-122). 96-well tissue culture plates (96-well, Greiner, 655160) were prepared by coating wells with 5 µg/mL anti-CD3 (OKT3, BioLegend, 317301). Select wells were co-coated with 20 µg/mL hVISTA-Fc by itself or pre-complexed with 1 µM anti-VISTA antibody (SG7, VSTB112, or isotype control). Proteins were coated in 50 µL PBS overnight, slowly rocking at 4 °C. Next, wells were washed twice with 200 µL PBS and 100,000 Jurkat T cells in 100 µL RPMI per well and incubated for 48 h at 37 °C. Cells were then washed twice with PBS + 1% BSA and analyzed by flow cytometry. BFP signal for each well condition was calculated using FlowJo software.

### Epitope mapping

A human VISTA library was created using error prone PCR to induce an average of one amino acid mutation per gene, as described previously^12^. The DNA library was electroporated into the EBY100 strain of *S. cerevisiae* yeast for a resulting library size of ~ 2*10^8^ mutants. Yeast displaying hVISTA mutants that lost binding to SG7 but retained binding to VSTB112 were isolated from the library using an alternating positive and negative FACS screening strategy, as described previously^12^. Yeast were incubated with the following concentrations of antibody: Sort 1, 500 pM SG7; Sort 2, 5 nM SG7; Sort 3, 100 nM VSTB112; Sort 4, 25 nM SG7. Yeast plasmid DNA from populations collected after Sorts 2–4 was amplified using Illumina MiSeq adapter primers and deep sequenced by Genewiz (EZ-Amplicon NGS). Reads were aligned to a template hVISTA FASTA file (bwa-mem), alignment file was indexed and sorted (SAMtools), nucleotides were counted at each position (bam-readcount), and amino acid mutation frequency was calculated (Biopython translate).

### Tumor studies

B16F10 cells (ATCC CRL-6475) and MC38 cells were cultured in Dulbecco’s Modified Eagle’s Medium (DMEM), supplemented with 10% FBS and 1% Pen-Strep. 4T1 cells (ATCC CRL-2539) were cultured in RPMI, supplemented with 10% FBS and 1% Pen-Strep. Cells were resuspended in cold PBS and injected in 100 µL volumes. 0.1 × 10^6^ B16F10 cells and 0.5 × 10^6^ MC38 cells were injected in the right flank of 8–10 week old female C57Bl/6J mice (The Jackson Laboratory, 000664). 0.5 × 10^6^ 4T1 cells were injected into the right flank of 8–10 week old female BALB/cJ mice (The Jackson Laboratory, 000651). In all cases, after reaching an average volume of 70–110 mm^3^, tumors were measured and mice were binned into treatment groups with equal mean tumor volumes. In all experiments, treatments were administered intraperitoneally. For B16F10 study, 10 mg/kg SG7-mIgG2a LALA/PG was given 2×/week for two weeks (total 4 injections). For MC38 study, 30 mg/kg SG7-mIgG2a LALA/PG and/or 5 mg/kg anti-PD-1 (Bio X Cell, Clone 29F.1A12) was given 2×/week for a total of 3 injections. For 4T1 study, 30 mg/kg SG7-mIgG2a or 30 mg/kg SG7-mIgG2a LALA/PG was given 2×/week for a total of three injections. Tumors were measured 2×/week with digital calipers. Animals were continuously monitored and were euthanized if tumors reached euthanasia criteria (1,500 mm^3^). Studies were concluded when more than one mouse in any treatment group reached euthanasia criteria.

### Flow cytometry analysis of immune infiltrates

Mice were euthanized by CO_2_ asphyxiation and tumors were manually extracted. Tumors were minced with a razor blade and resuspended in dissociation buffer: Hank’s balanced salt solution (HBSS) supplemented with 0.1 mg/mL Liberase TL (Sigma Aldrich, 05401020001) and 0.1 mg/mL DNAse I (Sigma Aldrich, 10104159001). Resuspended tumors were incubated in 15 mL conical tubes at 37 °C for 45 min, with gentle agitation every 15 min. Tumors were then passed through a 70 µM cell strainer (Fisher Scientific, 08-771-2) and red blood cells were lysed with ACK Lysis Buffer (Fisher Scientific, A1049201). Single cell suspensions were incubated with Fc block (BD Biosciences, 553142) and a fixable live/dead aqua stain (Thermo Fisher, L34957). Cells were then stained with a T cell panel: CD45, CD3, CD4, CD8 or a myeloid cell panel: CD45, CD11b, Ly6G, Ly6C, CD11c, and F4/80 of fluorescent antibodies. Stained cells were analyzed on a BD Biosciences LSR II flow cytometer (Stanford FACS Facility).

### Quantification and statistical analysis

All graphs and binding curve regressions were created using GraphPad Prism Version 8.0.2. The number of replicate samples in each experiment is specified in the figure legend. Error bars represent the standard deviation from the mean unless otherwise stated.

### Ethics statement

Mice were maintained and animal experiments performed in accordance with policies approved by the Stanford University Administrative Panel on Laboratory Animal Care (Protocol no. 33214) and conducted in accordance with Stanford University animal facility guidelines.

## Supplementary information

Supplementary file1

## Data Availability

No datasets were generated or analyzed during the current study.

## References

[1] Wang L (2011). VISTA, a novel mouse Ig superfamily ligand that negatively regulates T cell responses. J. Exp. Med..

[2] ElTanbouly MA (2020). VISTA is a checkpoint regulator for naïve T cell quiescence and peripheral tolerance. Science.

[3] Mercier IL (2014). VISTA regulates the development of protective antitumor immunity. Cancer Res..

[4] Gao J (2017). VISTA is an inhibitory immune checkpoint that is increased after ipilimumab therapy in patients with prostate cancer. Nat. Med..

[5] Xie S (2018). Expression of the inhibitory B7 family molecule VISTA in human colorectal carcinoma tumors. Cancer Immunol. Immunother..

[6] Kakavand H (2017). Negative immune checkpoint regulation by VISTA: A mechanism of acquired resistance to anti-PD-1 therapy in metastatic melanoma patients. Mod. Pathol..

[7] Liu J (2018). High-density infiltration of V-domain immunoglobulin suppressor of T-cell activation up-regulated immune cells in human pancreatic cancer. Pancreas.

[8] Villarroel-Espindola F (2018). Spatially resolved and quantitative analysis of VISTA/PD-1H as a novel immunotherapy target in human non-small cell lung cancer. Clin. Cancer Res..

[9] Lines JL (2014). VISTA is an immune checkpoint molecule for human T cells. Cancer Res..

[10] Flies DB (2014). Coinhibitory receptor PD-1H preferentially suppresses CD4+ T cell–mediated immunity. J. Clin. Invest..

[11] Flies DB, Higuchi T, Chen L (2015). Mechanistic assessment of PD-1H coinhibitory receptor-induced T cell tolerance to allogeneic antigens. J. Immunol. Baltim. Md.

[12] Mehta N (2019). Structure and functional binding epitope of V-domain Ig suppressor of T cell activation. Cell Rep..

[13] Wang J (2019). VSIG-3 as a ligand of VISTA inhibits human T-cell function. Immunology.

[14] Yang W (2017). Construction of a versatile expression library for all human single-pass transmembrane proteins for receptor pairings by high throughput screening. J. Biotechnol..

[15] Johnston RJ (2019). VISTA is an acidic pH-selective ligand for PSGL-1. Nature.

[16] Bradbury ARM, Sidhu S, Dübel S, McCafferty J (2011). Beyond natural antibodies: The power of in vitro display technologies. Nat. Biotechnol..

[17] Chao G (2006). Isolating and engineering human antibodies using yeast surface display. Nat. Protoc..

[18] Snyder, L. A., Powers, G., Ubani, E. Z. & Marvel, D. M. Anti-vista antibodies and fragments, uses thereof, and methods of identifying same. US Patent 20170320950 (2017).

[19] Mosely SIS (2017). Rational selection of syngeneic preclinical tumor models for immunotherapeutic drug discovery. Cancer Immunol. Res..

[20] Prodeus A (2020). VISTA.COMP—an engineered checkpoint receptor agonist that potently suppresses T cell–mediated immune responses. JCI Insight.

[21] Liu J, Yuan Y, Chen W, Putra J, Suriawinata AA, Schenk AD, Miller HE, Guleria I, Barth RJ, Huang YH, Wang L (2015). Immune-checkpoint proteins VISTA and PD-1 nonredundantly regulate murine T-cell responses. Proceedings of the National Academy of Sciences.

[22] Boder ET, Wittrup KD (2000). Yeast surface display for directed evolution of protein expression, affinity, and stability. Methods Enzymol..

[23] Van Deventer JA, Wittrup KD (2014). Yeast surface display for antibody isolation: library construction, library screening, and affinity maturation. Methods Mol. Biol. Clifton NJ.

[24] Colby DW (2004). Engineering antibody affinity by yeast surface display. Methods Enzymol..

[25] Van Deventer JA, Wittrup KD, Ossipow V, Fischer N (2014). Yeast surface display for antibody isolation: library construction, library screening, and affinity maturation. Monoclonal Antibodies: Methods and Protocols.

[26] Lo M (2017). Effector-attenuating substitutions that maintain antibody stability and reduce toxicity in mice. J. Biol. Chem..

