# Supplementary material for: An engineered antibody binds a distinct epitope and is a potent inhibitor of murine and human VISTA
Source: Sci Rep. 2020 Sep 16;10:15171. doi: 10.1038/s41598-020-71519-4 (PMC7494997; doi:10.1038/s41598-020-71519-4)
Supplement: Supplementary file 1 — Supplementary file1 [file 41598_2020_71519_MOESM1_ESM.pdf]

## Supplementary Information

# An engineered antibody binds a distinct epitope and is a potent inhibitor of murine and human VISTA

Nishant Mehta[1], Sainiteesh Maddineni[1], Ryan L. Kelly[2], Robert B. Lee[3], Sean A. Hunter[1,4], John L. Silberstein[1,5], R. Andres Parra Sperberg[1], Caitlyn L. Miller[1], Amanda Rabe[1,4], Louai Labanieh[1], Jennifer R. Cochran[1,2,3,4,5*]

[1]Department of Bioengineering, Stanford University, Stanford, CA 94305, USA
[2]xCella Biosciences, Menlo Park, CA 94025, USA
[3]Department of Chemical Engineering, Stanford University, Stanford, CA 94305, USA
[4]Cancer Biology Program, Stanford University School of Medicine, Stanford, CA 94305, USA
[5]Immunology Program, Stanford University School of Medicine, Stanford, CA 94305, USA

*To whom correspondence should be addressed: jennifer.cochran@stanford.edu

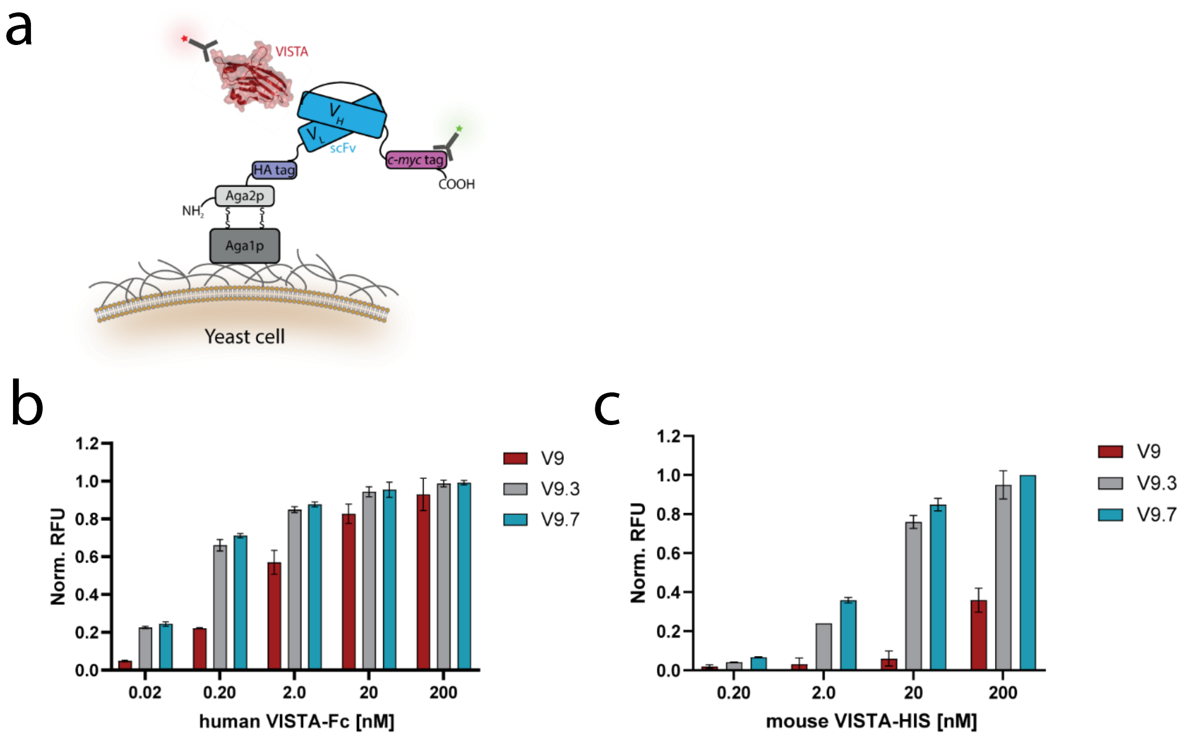


**Supplementary Figure S1: Yeast display schematic and binding analysis of scFv clones**. **(a)** Schematic used for scFv display and screening on yeast. Soluble mouse and human VISTA were used as screening antigens for scFv mutants individually fused to the yeast Aga2p mating protein and displayed on the cell surface. Fluorescent antibodies against a C-terminal c-myc epitope tag or VISTA were used to detect expression levels and binding, respectively. **(b)** Binding assays of scFv clones V9, V9.3, and V9.7 displayed on yeast with serial dilutions of hVISTA-Fc antigen or **(c)** serial dilutions of mVISTA-His antigen. Affinity matured clones V9.3 and V9.7 show significant improvement in VISTA binding over parent clone V9 isolated from the initial yeast sFv library. Mean ± standard deviation of triplicate measurements are shown.

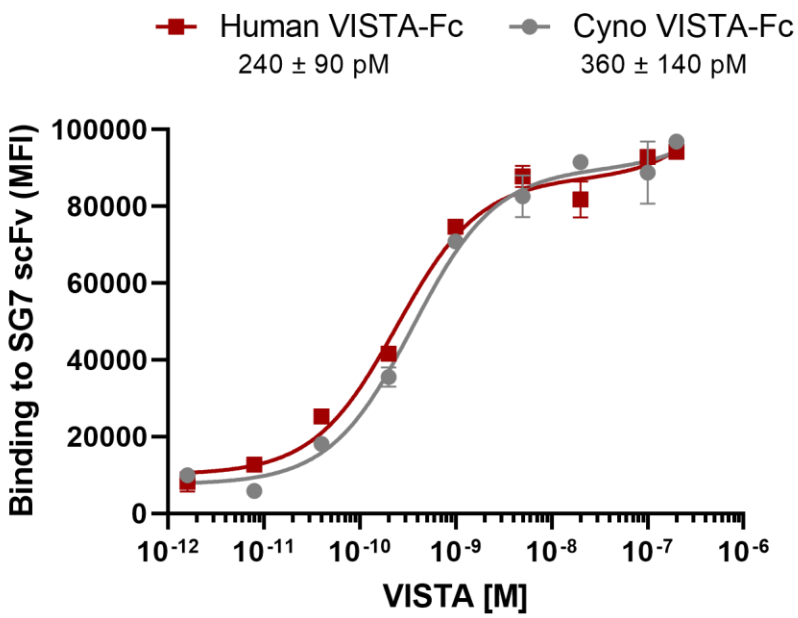


**Supplementary Figure S2: Binding of SG7 scFv to human and cyno VISTA-Fc.** SG7 displayed in scFv form on the surface of yeast was incubated with serial dilutions of cyno VISTA-Fc or human VISTA-Fc. The apparent binding affinities show that there is strong binding (< 0.5 nM) affinity to both species. Mean ± standard deviation of duplicate measurements are shown.

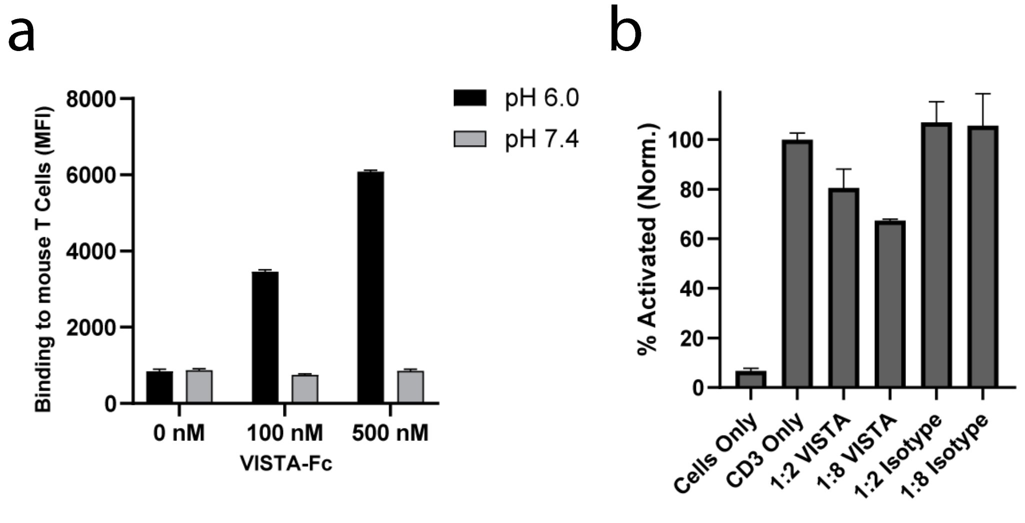


**Supplementary Figure S3: Optimization of T cell binding and activation assays. (a)** Binding of three different concentrations of VISTA-Fc was measured with activated mouse T cells at pH 6.0 and pH 7.4 Dose-responsive binding was observed at pH 6.0 while no significant binding was seen at pH 7.4. Mean ± SD of duplicate measurements are shown. **(b)** Jurkat NFAT-BFP reporter cells were incubated in the presence of anti-CD3 only, anti-CD3 with hVISTA-Fc, or anti-CD3 with an Fc fusion isotype control. A weight ratio of 1:2 or 1:8 of anti-CD3:hVISTA-Fc or an anti-CD3:isotype control was used. The presence of hVISTA-Fc prevented full T cell activation, whereas the Fc fusion isotype control did not change activation levels. Mean ± standard deviation of triplicate measurements are shown.

a

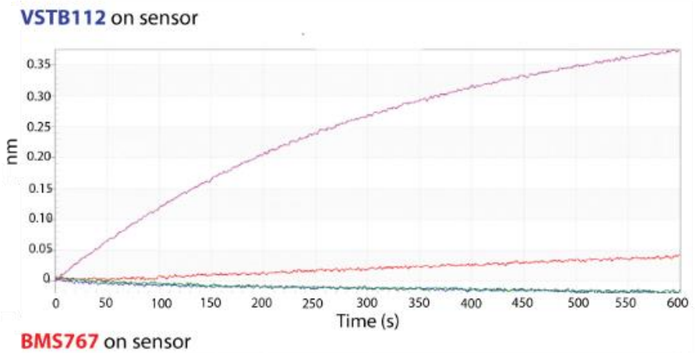


| | Secondary Ab | Analyte Conc. (nM) | % Response | Block/Pair? |
|---|---|---|---|---|
| | VSTB112 | 20 | -4.55% | Block |
| | BMS767 | 20 | 10.37% | Block |
| | SG7 | 20 | -4.15% | Block |
| | VISTA Ag Only | 20 | 100.00% | N/A |

b

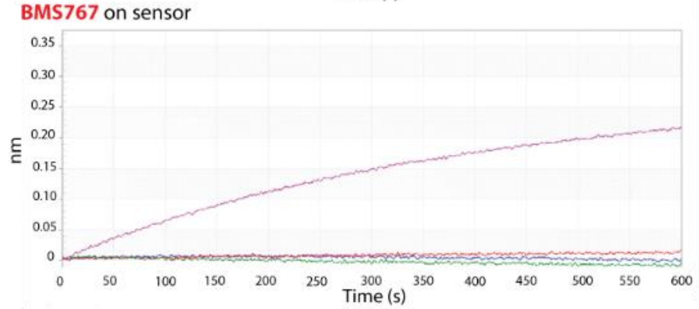


| | Secondary Ab | Analyte Conc. (nM) | % Response | Block/Pair? |
|---|---|---|---|---|
| | VSTB112 | 20 | 0.60% | Block |
| | BMS767 | 20 | 5.98% | Block |
| | SG7 | 20 | -3.20% | Block |
| | VISTA Ag Only | 20 | 100.00% | N/A |

c

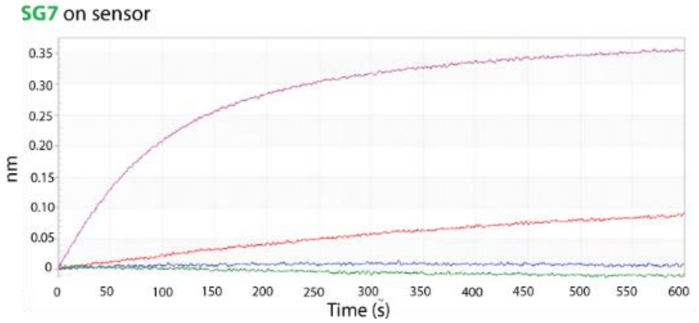


| | Secondary Ab | Analyte Conc. (nM) | % Response | Block/Pair? |
|---|---|---|---|---|
| | VSTB112 | 20 | 2.36% | Block |
| | BMS767 | 20 | 24.78% | Block |
| | SG7 | 20 | -2.70% | Block |
| | VISTA Ag Only | 20 | 100.00% | N/A |

**Supplementary Figure S4: Epitope binning of VISTA antibodies**. The potential for overlapping antibody epitopes was measured with a ForteBio Octet. The amount of secondary antibody that could bind to an adhered primary antibody was measured with bio-layer interferometry. The first antibody was adhered to the optical biosensor, human VISTA-Fc was loaded ('load' step), and then all other antibodies or VISTA antigen were associated to the loaded sensor ('association' step). The signal read out from the association step for **(a)** VSTB112, **(b)** BMS767, and **(c)** SG7 adhered to the sensor is shown here. The results table for each loaded sensor shows response in wavelength change (nm) as well as percentage change (% response) from baseline. Only VISTA-Fc (VISTA Ag) signal significantly increases, demonstrating the lack of binding from all other antibodies once VISTA is bound to the adhered antibody on the sensor. All three antibodies can therefore block each other from binding VISTA.

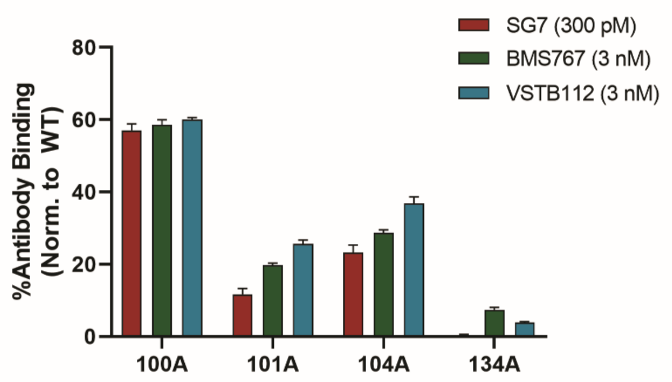


**Supplementary Figure S5: Mutational analysis of buried residues in the VISTA extracellular domain.** Four residues that were either buried within the beta barrel (M101, L104, L134) or on the opposite side of the predicted epitope (T100) were expressed as individual point mutations by yeast surface display. Binding to all three antibodies was partially reduced (<75%) for T100A and significantly reduced (<50%) for M101A, L104A, L134A. Binding to VSTB112 compared to SG7 was slightly higher for 101A, 104A, and 134A, partially explaining how these clones came through the negative and positive sorts. Based on the partial binding decrease, T100A is predicted to destabilize the whole protein but not be specific to any particular epitope. Mean ± standard deviation of triplicate measurements are shown.

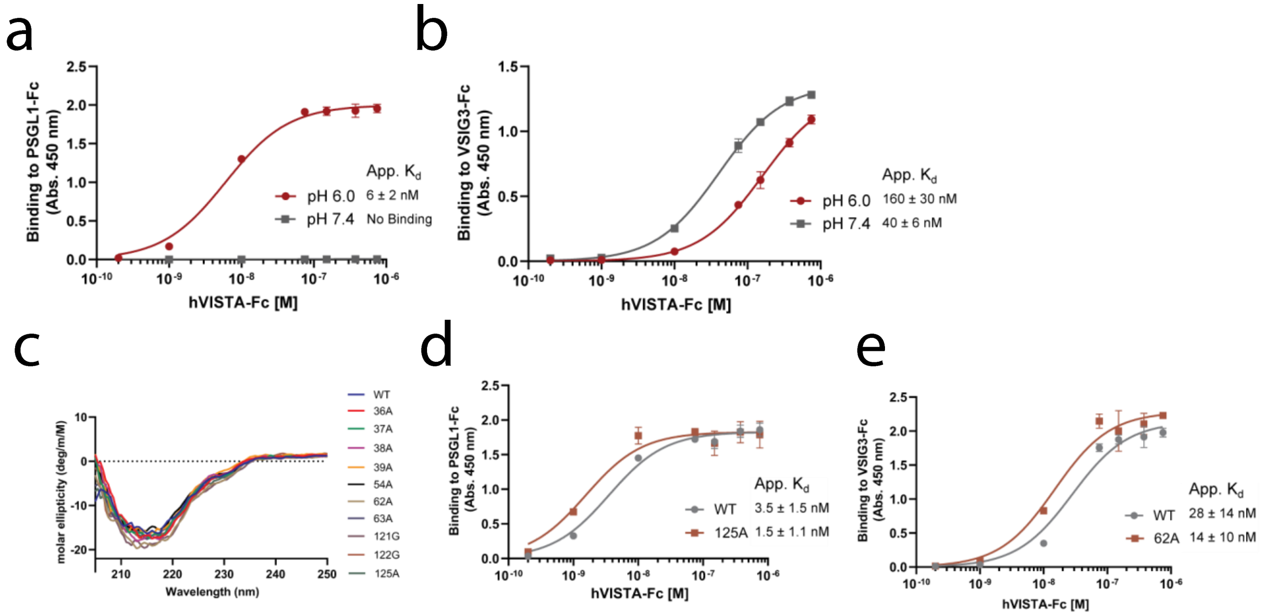


**Supplementary Figure S6: Binding of soluble hVISTA to PSGL-1 and VSIG3. (a)** Binding measurements of soluble hVISTA-Fc to microtiter well-coated PSGL-1-Fc at pH 6.0 or 7.4. No detectable binding is observed at pH 7.4. **(b)** Binding measurements of soluble hVISTA-Fc to microtiter well-coated VSIG3-Fc at pH 6.0 or 7.4. Binding at physiological pH is about four times stronger than binding at pH 6.0. **(c)** Circular dichroism spectra of soluble hVISTA mutants. No significant aberrations in the curves are observed, providing evidence that mutations do not cause significant changes in secondary structural elements. **(d)** Binding of wild-type hVISTA-Fc or 125A-hVISTA-Fc to microtiter well-coated PSGL-1-Fc. **(e)** Binding of wild-type hVISTA-Fc or 62A-hVISTA-Fc to microtiter well-coated VSIG3-Fc. Mean ± standard deviation of duplicate measurements are shown.

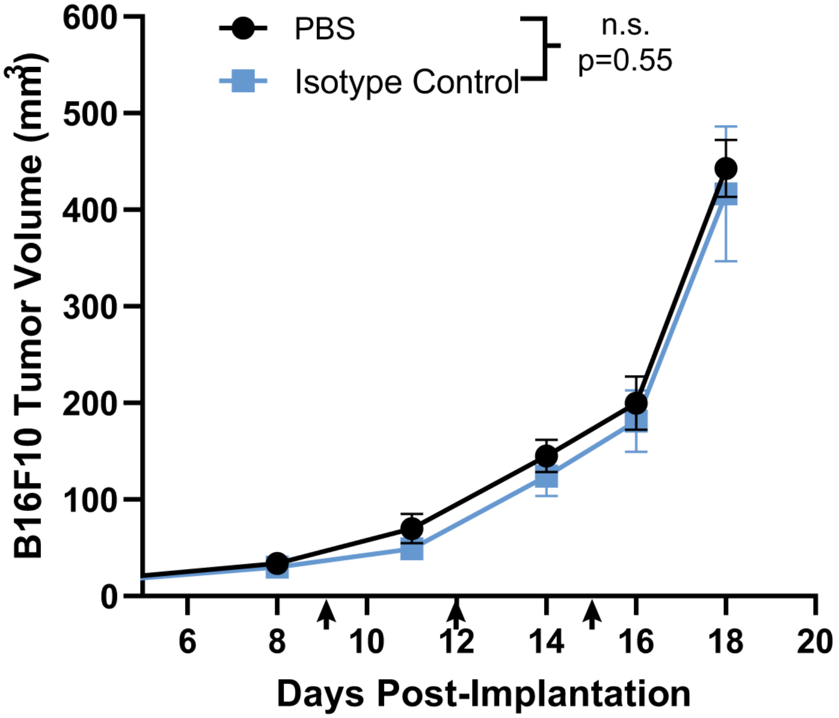


**Supplementary Figure S7: Comparison of PBS and antibody isotype control in B16F10 tumor model**. There is no significant difference between PBS and a mIgG2a antibody isotype control on the growth of B16F10 tumors; n=5 mice per group, Mean ± SEM are shown, p-values calculated by two-way ANOVA. B16F10 tumors were treated with 100 µL PBS or 10 mg/kg mIgG2a isotype control starting on day 9 post-implantation (black arrows).

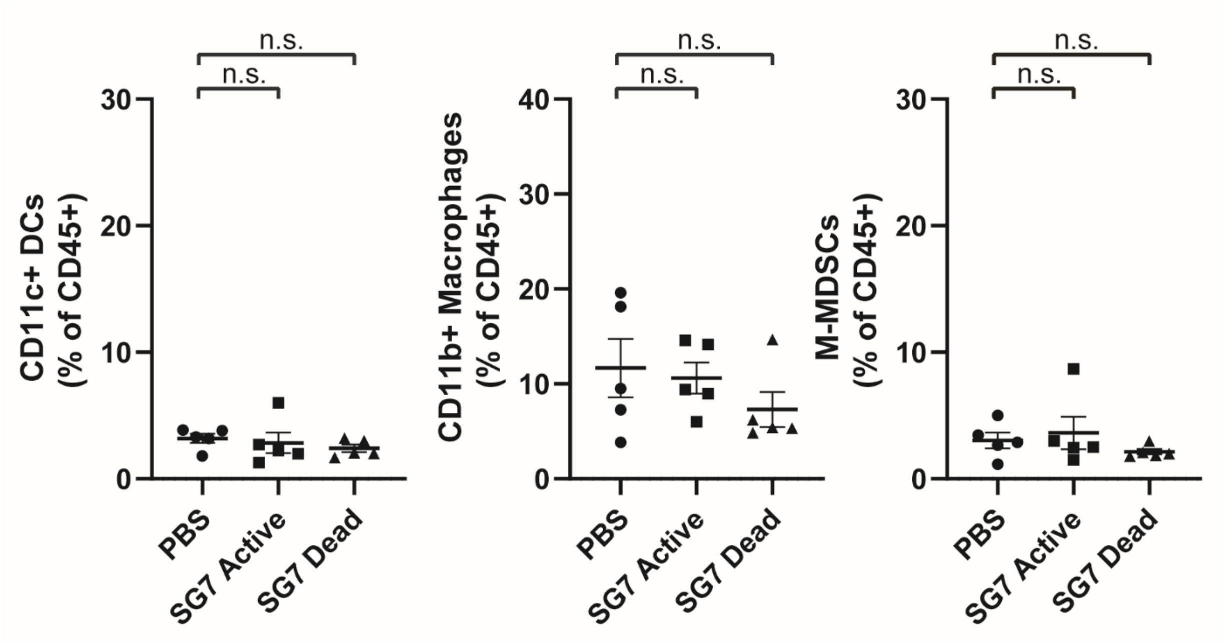


**Supplementary Figure S8: Effect of SG7 on additional myeloid cell subsets.** Immune flow analysis of extracted 4T1 tumors on Day 17. The percentage of CD11c+ dentritic cells, CD11b+ macrophages, and M-MDSCs out of total CD45+ cells in each tumor sample are shown. Mean ± SEM are shown. P-values calculated by one-way ANOVA (DMCT), n.s. = p>0.05.

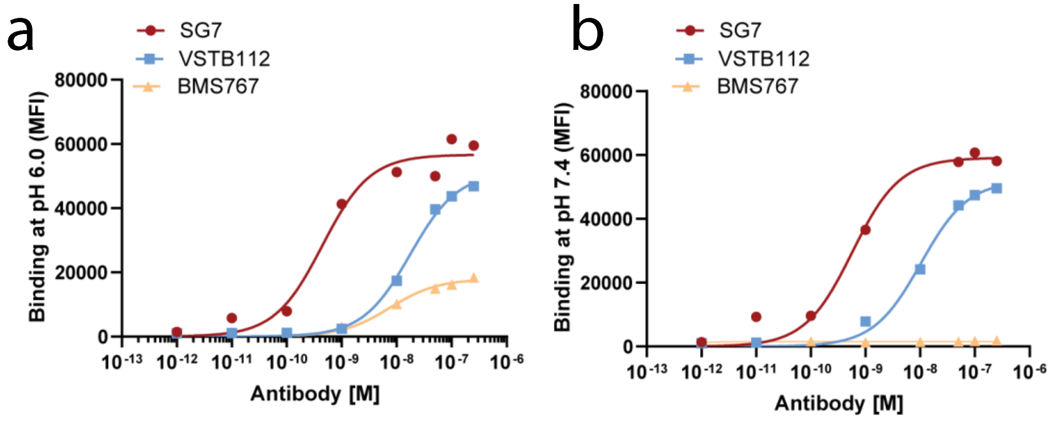


c

| | pH 6.0 | pH 7.4 |
|---|---|---|
| **SG7** | 0.4 ± 0.3 nM | 0.6 ± 0.4 nM |
| **VSTB112** | 17 ± 5 nM | 10 ± 5 nM |
| **BMS767** | 7 ± 5 nM | No Binding |

**Supplementary Figure S9: pH dependence of SG7, VSTB112, BMS767 binding to hVISTA displayed on yeast. (a)** Binding of antibody to hVISTA extracellular domain displayed on yeast at pH 6.0. All three antibodies bind under this condition. **(b)** Binding of antibody to hVISTA extracellular domain displayed on yeast at pH 7.4. The SG7 and VSTB112 antibodies still bind strongly but BMS767 has negligible binding at physiological pH. **(c)** Table of apparent $K_d$ values for each antibody interaction. SG7 has significantly stronger affinity as compared to VSTB112 and BMS767.
